# Meiosis in an asymmetric dikaryotic genome of *Tremella fuciformis* Tr01 facilitates new chromosome formation

**DOI:** 10.1186/s13059-023-03093-7

**Published:** 2023-12-05

**Authors:** Youjin Deng, Lin Guo, Longji Lin, Yuefeng Li, Jinxiang Zhang, Yue Zhang, Bin Yuan, Lina Ke, Baogui Xie, Ray Ming

**Affiliations:** 1 College of Life Science, Fujian Agriculture and Forestry University, Fuzhou, Fujian 350002 China; 2 Center for Genomics, Fujian Agriculture and Forestry University, Fuzhou, Fujian 350002 China; 3 Zhangzhou Institute of Agricultural Science, Zhangzhou, Fujian 363005 China; 4 Department of Plant Biology, University of Illinois at Urbana-Champaign, 1201 W. Gregory Drive, Urbana, IL 61801 USA

**Keywords:** Centromere-free, Dikaryotic, Inter-chromosomal recombination, New chromosome formation, Sporeless

## Abstract

**Background:**

The dikaryotic stage dominates most of the life cycle in basidiomycetes, and each cell carries two different haploid nuclei. Accurate phasing of these two nuclear genomes and their interactions have long been of interest.

**Results:**

We combine PacBio HiFi reads, Nanopore ultra-long reads, and Hi-C data to generate a complete, high-quality asymmetric dikaryotic genome of *Tremella fuciformis* Tr01, including Haplotypes A and B genomes. We assemble a meiotic haploid DBZ04 genome and detect three recombination events in these two haplotypes. We identify several chromosomal rearrangements that lead to differences in chromosome number, length, content, and sequence arrangement between these two haplotypes. Each nucleus contains a two-speed genome, harboring three accessory chromosomes and two accessory compartments that affect horizontal chromatin transfer between nuclei. We find few basidiospores are ejected from fruiting bodies of Tr01. Most monospore isolates sequenced belong to Tr01-Haplotype A genome architecture. More than one-third of monospore isolates carry one or two extra chromosomes including Chr12B and two new chromosomes ChrN1 and ChrN2. We hypothesize that homologous regions of seven sister chromatids pair into a large complex during meiosis, followed by inter-chromosomal recombination at physical contact sites and formation of new chromosomes.

**Conclusion:**

We assemble two haplotype genomes of *T. fuciformis* Tr01 and provide the first overview of basidiomycetous genomes with discrete genomic architecture. Meiotic activities of asymmetric dikaryotic genomes result in formation of new chromosomes, aneuploidy of some daughter cells, and inviability of most other daughter cells. We propose a new approach for breeding of sporeless mushroom.

**Supplementary Information:**

The online version contains supplementary material available at 10.1186/s13059-023-03093-7.

## Background

Genomic variations enable organisms to adapt to changing environment and are the basis for natural selection. Chromosomal rearrangements, a type of large-scale genomic variation, mediate genomic macroevolution [1]. Rearrangements can occur within a chromosome, or across different chromosomes, referring to intra- and inter-chromosomal rearrangements, respectively. With advances in long-read DNA sequencing, more chromosomal rearrangements were uncovered in fungi.

Chromosomal rearrangements can play an important role in cellular processes. For example, aneuploidy and isochromosome formation in pathogenic *Candida albicans* can increase the copy number and transcript expression of key genes for fluconazole resistance [2]. Such resistance was also found in a related species, *Candida glabrata* [3], in which segmental duplications and new chromosome formation were found. In the fungal pathogen *Cryptococcus neoformans*, DNA double-stranded breaks (DSBs) in centromeric retrotransposons can lead to the formation of multiple inter-chromosomal rearrangements and new telomeres [4]. The resulting strains containing chromosome translocations can fail to undergo sexual reproductions with the parental genotype. Centromere-mediated chromosome rearrangements have also been detected in *C. amylolentus* [5], *Candida* [6], and *Malassezia* [7]. Chromosome rearrangements are mediated by many different mechanisms, such as meiotic recombination [8], transposable elements [9], and repair of DSBs during meiosis [4].

*Tremella fuciformis* is also known as the white jelly mushroom, snow ear, and silver ear fungus and belongs to the Tremellaceae in the order Tremellales. This edible fungus is a popular food in East Asia for its translucent, leafy, white or pale yellow fruiting bodies. This species has also been useful for cosmetic and medicinal purposes due to its diverse anti-aging, immunomodulatory, memory improving, and antitumor physiological activities [10–13]. *T. fuciformis* is a widespread fungus easily found in the tropical and subtropical areas and has been developed as one of the highly popular cultivated mushrooms in China and other countries in East Asia. In all environments including cultivation conditions, *T. fuciformis* requires the presence of another fungus, *Annulohypoxylon stygium* in the family Xylariaceae in the Ascomycetes, to assist its growth and reproduction [14]. *T. fuciformis* is dimorphic, in that it can exist as yeast-like cells that reproduce asexually by budding, or as filamentous cells that grow by apical extension. Only the filamentous cells can build connections with *A. stygium* cells and grow into fruiting bodies. Tr01 is a common strain of *T. fuciformis* widely cultivated in China, forming white fruiting bodies. One distinguishing feature of this strain is that it produces few basidiospores.

For many fungi belonging to the basidiomycetes, such as *T. fuciformis*, sexual compatibility is determined by two genetically unlinked mating type (*MAT*) loci in a so-called tetrapolar mating system. One of the *MAT* loci encodes pheromone receptors (*P/R*) and pheromone precursors, whereas the other locus harbors genes encoding homeodomain (*HD*) transcription factors [10]. Two monokaryotic individuals with different alleles at both *MAT* loci are able to undergo plasmogamy and form a dikaryotic strain. The dikaryotic stage dominates the life cycle in which each individual carries two different haploid nuclei [11]. Under the appropriate conditions, dikaryotic mycelia grow into fruiting bodies, undergo plasmogamy and meiosis, and generate basidia, each of which produces four basidiospores.

We combined PacBio HiFi reads, Nanopore ultra-long reads as well as Hi-C data to generate two haplotype genomes of *T. fuciformis* dikaryotic strain Tr01. Genome-wide sequence analyses were performed to detect possible discrepant genome architectures within the nucleus. Two nuclear genomes were then compared to discover possible inter-nuclear chromosomal arrangements. Dozens of monospore strains were isolated and sequenced to analyze the possible meiotic behaviors of this dikaryotic genome. We have proposed a hypothesis to explain the abnormal meiotic products derived from this asymmetric dikaryotic genome. Our studies aim to understand the meiotic behavior of an asymmetric dikaryotic genome and to develop new approaches for sporeless mushroom breeding.

## Results

### Genomic assemblies of monokaryotic isolate DBZ04 and dikaryotic isolate Tr01

DBZ04 was a monokaryotic isolate originated from the germination of a single basidiospore of *T. fuciformis* dikaryotic Tr01. A gap-free genome of DBZ04 was assembled using PacBio long reads combined with high-throughput chromosome conformation capture (Hi-C) data [12]. The 27.94 Mb genome was organized 11 chromosomes (Additional file 1: Fig. S1) and a circular mitochondrial genome was also assembled. The chromosomes varied in length, with the largest at 8.62 Mb and the smallest at less than 1 Mb (Additional file 1: Fig. S1). The end of each chromosome contained 20–40 tandem telomeric repeats (TTA(G)_3–5_). An rDNA cluster located at 2.76–2.90 Mb in Chr02 contained approximately 16 tandem rDNA unit repeats of a total of 8394 bp (Additional file 1: Fig. S2).

One potential centromere region (40 kb per region) for each chromosome was predicted by Hi-C mapping (Additional file 1: Fig. S3). Each region was unique but all were rich in repeat elements, occupying 69.5 to 100% of whole regions (Additional file 2: Table S1). All regions except for that of Chr10 contained at least two copies of Tcn6 transposon, except for those of Chr01 and Chr10 contained at least one copy of Tcn1 transposon. Both Tcn1 and Tcn6 transposons are commonly found in centromeres of *Cryptococcus* species complex [13], which belong to Tremellaceae, in the same order with *T. fucifomis*. Rnd-4 family-1813, belonging to the Ty1-*copia* family of retroelements, was also found in most centromere regions of DBZ04 genome.

PacBio circular consensus sequencing of Tr01 yielded 211,688 HiFi reads adding up to 3.26 Gb of data representing appropriately 60× coverage of the dikaryotic genomes. 40.3 Gb (713×) Oxford Nanopore ultra-long read sequences of Tr01 genome were generated and 31,712 Nanopore ultra-long reads larger than 100 kb, totaling 4.04 Gb (72×), were selected to assist and verify Tr01 genome assembly. HiFiasm in Hi-C mode assembled the HiFi reads into two sets of phased contigs. One set was 30.3 Mb in size containing five complete chromosomes, and the other set was 30.2 Mb in size with seven complete chromosomes. Analyses of rDNA region in each genome set linked rDNA-related contigs into one telomere to telomere (T to T) chromosome. Polished Nanopore ultra-long reads further were used to bridge gaps and assembled the rest 41 contigs of first genome set into six complete chromosomes, and the rest 46 contigs of second genome set into three complete chromosomes using telomeric repeat sequences as markers to reach T to T chromosome assemblies. After phased by *NuclearPhaser* [14], 11 T to T chromosomes were clustered into Tr01-Haplotype A genome, and the other 12 T to T chromosomes in Tr01-Haplotype B genome. For chromosomes with heterozygosity between two haplotype genomes, each chromosome had significantly stronger Hi-C contact signals with chromosomes from same nucleus than those from the other nucleus (*P*-value = 3.9E-12 in one-way ANOVA, Additional file 1: Fig. S4).

Nanopore ultra-long reads were mapped to the Haplotype A and B genomes to verify the assembly. There were 7 TE insertions and 24 TE deletions in Haplotype A genome, and 5 TE insertions and 18 TE deletions in Haplotype B genome (Additional file 2: Table S2). These TEs were mainly composed of 16 copies of 683 bp LTR (30%), 16 copies of 1239 bp LTR (30%), and 7 copies of 6550 bp LTR (13%). HiFi reads were mapped across each of the TE InDels and verified that the sequences in the haplotypes A and B were correct, and all TE InDels. For example, two tandemly duplicated 6587 bp TEs were mapped and verified by 45 HiFi reads cross these two TEs, but the Nanopore Ultra-long reads mapped to this region contained three tandemly duplicated 6587 bp TEs. Samples for PacBio sequencing and Nanopore sequencing were collected in 2021 and 2023, respectively, 18 months apart. TE InDels occurred during this period, separated by 12 rounds of vegetative reproduction, reflecting TE movement during mitoses in these 18 months. No chimeric contigs in Haplotypes A and B were detected, resulting from high accuracy and high redundancy of HiFi reads generated.

**Fig. 1:**
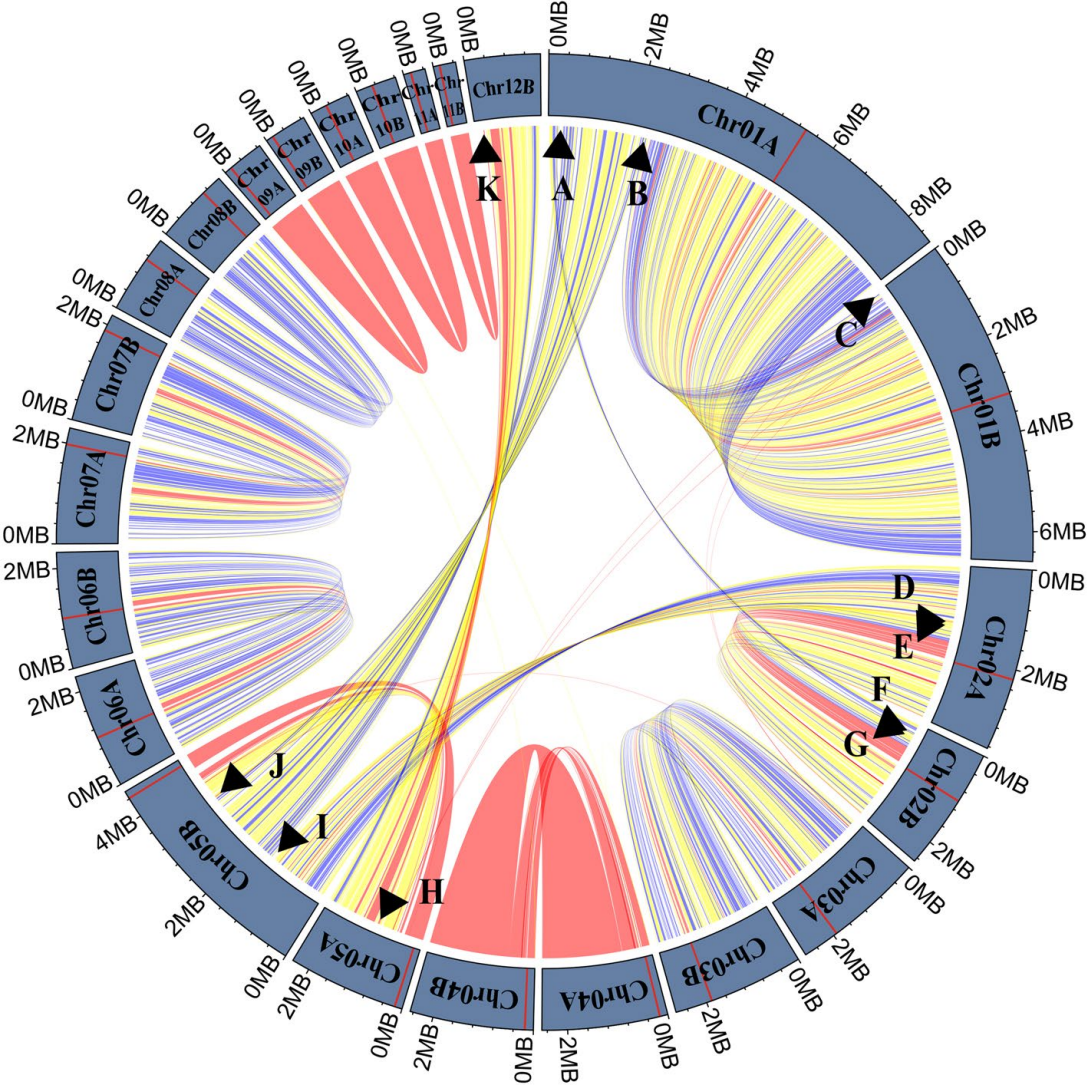
Linkage of similar sequences between the dikaryotic genomes (> 10 kb sequence) of *Tremella fuciformis*. Red bars in outer ring represent centromere locations of each chromosome. Red lines, ≥ 99% identity; yellow lines, ≥ 95% and < 99% identity; blue lines, ≥ 90% and < 95% identity; gray lines, < 90% identity. Black arrowheads mark breakpoints of structural variation between the dikaryotic genomes

**Fig. 2:**
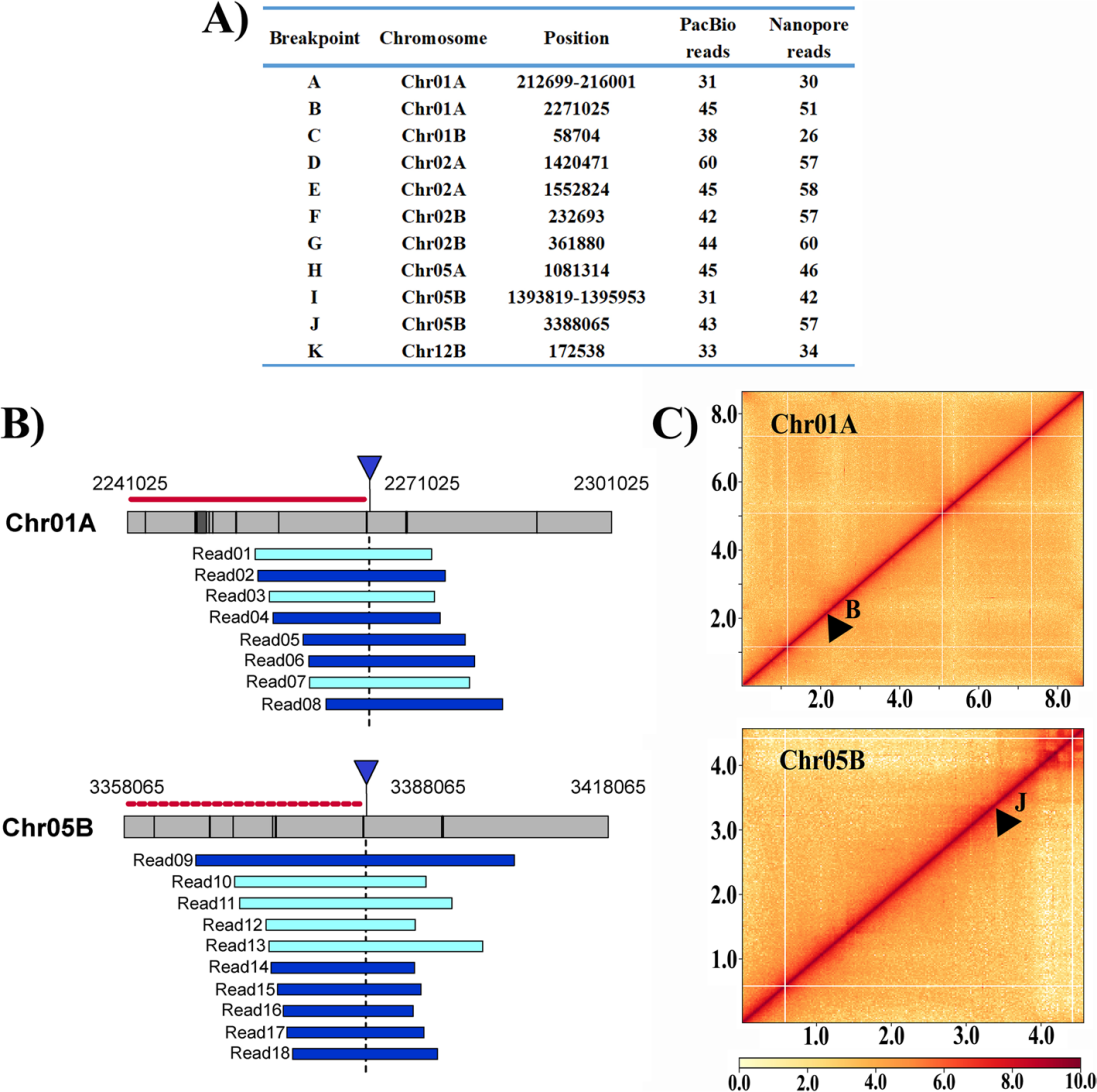
Verification of structural variations by PacBio HiFi and Nanopore ultra-long reads, and Hi-C data. **A** Number of PacBio HiFi and Nanopore ultra-long reads crossing the regions between 2-kb upstream and downstream of the breakpoints. All Nanopore ultra-long reads are larger than 100 kb in size. **B** Schematic diagram of long HiFi reads crossing the breakpoints B and J. Inverted triangles point to the breakpoints. Dark colors in the bars represent repeat sequence regions. Red solid lines and red dotted lines represent a set of homologous regions between the dikaryotic genomes. Blue bars represent reads mapped and light green bars represent reads mapped by reverse complementarity, respectively. **C** Heat map of Hi-C data supporting neighbor relationships between upstream and downstream sequences of breakpoints B and J. Black arrowheads mark breakpoints of chromosome rearrangement

The Tr01-Haplotype B genome of *T. fuciformis* Tr01 contained 12 chromosomes, one more than those in the Tr01-Haplotype A genome. Further, the sequences flanking six breakpoints in the Tr01-Haplotype B genome showed non-collinear arrangement with the Tr01-Haplotype A genome, revealing five structural variants between these two nuclear genomes, two each in Chr01A and Chr02A and one in Chr05A (Fig. 1). Both PacBio HiFi reads and Nanopore ultra-long reads (> 100 kb) supported the linkage between the upstream and downstream sequences (Fig. 2A) flanking each breakpoint in these structural variants. For example, the upstream sequences flanking breakpoints B and J were a pair of homologous sequences, but their downstream sequences had no similarity, indicating that structural variations in the form of homologous recombination took place at those sites. We mapped 45 and 43 long HiFi reads uniquely to sequences around breakpoints B and J, respectively, verifying the linkage of these structural variants (Fig. 2B). Strong Hi-C signals between upstream and downstream sequences of each breakpoint (B and J shown in Fig. 2C, others shown in Additional file 1: Fig. S5) also supported their neighbor relationship. For both Tr01-Haplotypes A and B genomes, each end of a chromosome showed strong contact signals with other chromosomal ends (Additional file 1: Fig. S6). The telomere-telomere contacts provided another evidence for genome assembly verification.

### Genomic comparison between DBZ04 and Tr01

All 11 chromosomes shared high sequence collinearity between Tr01-Haplotypes A and DBZ04 genome. Sequence similarity between these two genome, except for DBZ04 regions of Chr01:5177740-6011183, Chr05:1881594-2385495(3’end), Chr06 and Chr07:1662638-2272366(3’end), were higher than 99.99%, whereas the four sequences showed more than 99.99% identity with the corresponding ones in Tr01-Haplotypes B genome. As a result, four homologous recombination breakpoints occurred in DBZ04 genome during meiosis, which carried ~82% and ~18% of Tr01-Haplotypes A and B source sequences, respectively (Additional file 1: Fig. S7). The recombination breakpoints were also verified by mapping of Nanopore ultra-long reads (Additional file 2: Table S3). Due to both genomes being generated independently, DBZ04 genomic sequences provided another evidence to verify the assembly of Tr01 genomes.

### Genomic comparison of two nuclei in *T. fuciformis* Tr01

Approximately half of the *T. fuciformis* Tr01 assemblies were assigned to either Tr01-Haplotype A or B genome, which are 28.15 and 28.34 Mb in length, respectively. Eight chromosomes shared high sequence collinearity between Tr01-Haplotypes A and B, including Chromosomes 3, 4, and 6 to 11. Three chromosomes contained rearrangements and sequence variation, including chromosomes 1, 2, and 5 between the two haplotypes (Fig. 1 and Table 1), resulting in different genomic architectures. Tr01-Haplotype A or B architectures were referred to as, based on sequence arrangements of their Chr01, Chr02, and Chr05, respectively. The additional Chr12B in the Tr01-Haplotype B genome was a centromere-free chromosome of 1.51 Mb, most of which was homologous to the right arm of Chr05A. Sequence alignments between the two haplotype genomes revealed four large orphan regions (> 50 kb), including Chr05A:795,575-913,748 and Chr07A:1-125,423 in the Tr01-Haplotype A genome, and Chr01B:1-58,696 and Chr12B:1-172,532 in Tr01-Haplotype B genome. Annotation of the Tr01-Haplotypes A and B genomes predicted 8480 and 8563 genes, including 100 and 134 haplotype-specific genes, respectively. 28.2% of the haplotype-specific genes were distributed in the orphan regions.

**Table 1:** Chromosome number, size, and gene content of *Tremella fuciformis* Tr01-Haplotype A and B nuclear genomes

Chromosome ID	Size (Mb)		Gene number	
	Haplotype A	Haplotype B	Haplotype A	Haplotype B
Chr01	8.65	6.59	2838	2132
Chr02	3.63	2.50	1097	767
Chr03	2.67	2.64	823	833
Chr04	2.49	2.41	683	676
Chr05	2.36	4.56	713	1372
Chr06	2.30	2.32	678	689
Chr07	2.33	2.21	729	712
Chr08	1.58	1.52	509	495
Chr09	0.86	0.85	142	142
Chr10	0.83	0.81	188	186
Chr11	0.43	0.42	80	76
Chr12		1.51		483
Total	28.15	28.34	8480	8563

A total of 0.6 million SNPs (average 21/kbp) were called between the two nuclear genomes of *T. fuciformis* Tr01, distributed mainly on Chr01, Chr02, and Chr03, and on Chr05, Chr06, Chr07, and Chr08 (Additional file 1: Fig. S8). Genome comparison predicted 2201 structural variants (SVs), including 1154 insertions in Tr01-Haplotype A and 1047 in Tr01-Haplotype B. These SVs exhibited a wide range of lengths from 21 to 32,759 bp and account for 4.5 and 7.1% of the corresponding genome size. Both nuclear genomes had similar proportions (~19%) of repetitive sequences, of which 70% were long terminal repeats (LTRs). Interestingly, two species-specific LTRs, LTR683 and LTR1239, were widely and unevenly distributed in the nuclei (Additional file 1: Fig. S9). A total number of 181 full-length LTR683s were detected in Tr01-Haplotype A, ~6 times the number in Tr01-Haplotype B. Inversely, 221 copies of full-length LTR1239s were found in the Tr01-Haplotype B genome, but just 4 in Tr01-Haplotype A. LTR683 and LTR1239 had a strong bias to Tr01-Haplotype A and B nuclei, respectively.

Homologous chromosomes of Chr04, Chr09, Chr10, and Chr11 were remarkably similar, with similarity of 99.99%, 100%, 100%, and 99.97%, respectively (Additional file 2: Table S4). However, multiple SVs were detected between each pair of the homologous chromosomes, accounting for 0.5 to 4.4% of the sequence size, and reaching density of 0.29 to 1.04 SVs per 100 kb. LTR683s were only detected in Chr09A, Chr10A, and Chr11A, and 4 times in Chr04A than in Chr04B. Inversely, LTR1239s were only detected in Chr10B and Chr11B, and 5 times in Chr04B than in Chr04A (Additional file 1: Fig. S9). Both LTRs were useful markers to distinguish these homologous chromosomes of low heterozygosity.

Similar to *T. mesenterica* [15], *T. fuciformis* is a tetrapolar basidiomycete. The two mating type loci in *T. fuciformis* Tr01 were not genetically linked to either the Tr01-Haplotype A or B genome. The pheromone and pheromone receptor (*P/R*) loci were located on Chr08, whereas both homeodomain (*HD*) loci were on Chr07. Polymorphic regions in the *P/R* loci were about 18 or 23 kb in length and harbored eight genes, seven of which showed allelic differences. Only 1–2-kb regions in the *HD* loci were polymorphic, including partial sequences of *SXI1* and *SXI2* genes, as well as their intergenic region (Additional file 1: Fig. S10).

### Two-speed genome of *T. fuciformis* Tr01

**Fig. 3:**
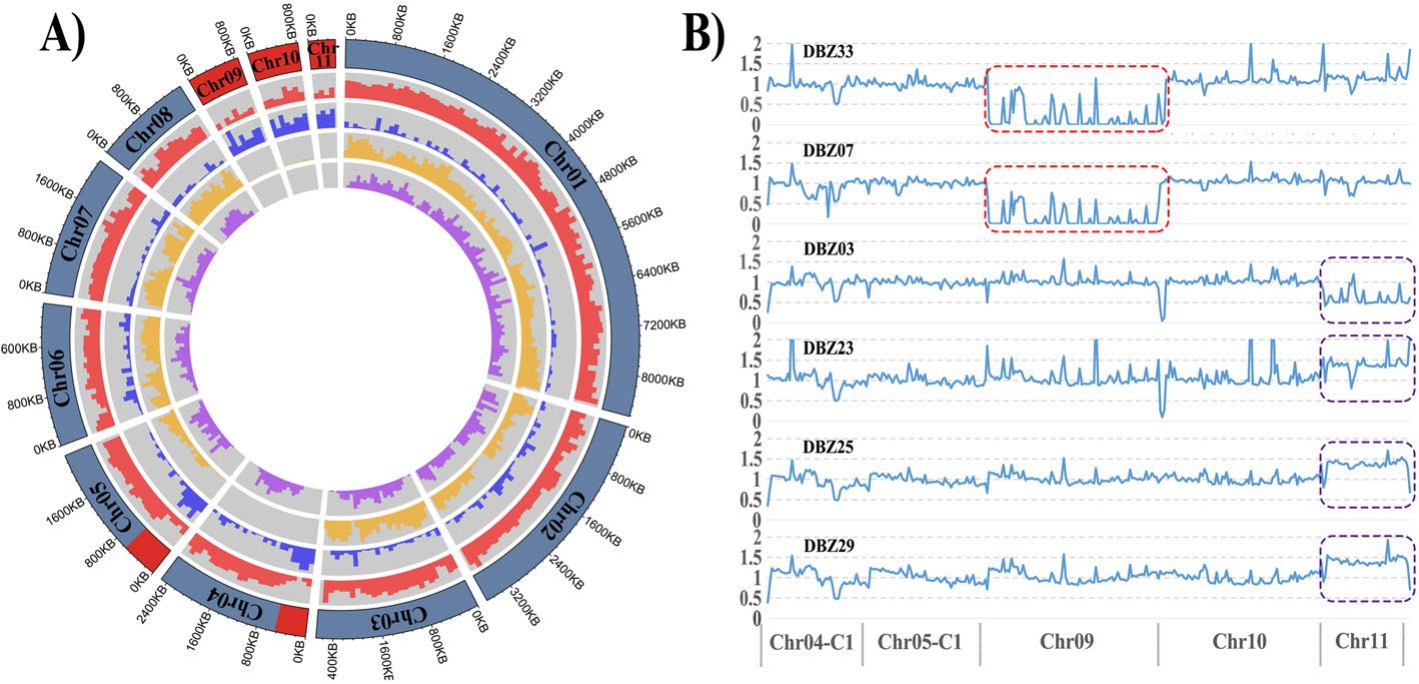
Features of dikaryotic genomes in Tr01. **A** Circos representation of the dikaryotic genome of Tr01-Haplotype A. From outer to inner layer: (1) distribution of core (blue) and accessory (red) chromosomes/compartments in the Tr01-Haplotype A genome; (2) gene density in 100-kb windows; (3) percentage of repetitive sequences in 100-kb windows; (4) SNP density between Tr01-Haplotype A and B genomes in 100-kb windows; (5) distribution of BUSCOs. **B** Aneuploid accessory regions of single-basidiospore isolates. For each isolate, normalized read depth in 10-kb windows is shown along accessory regions relative to the Tr01-Haplotype A genome. Copy number variations of Chr09 are shown in red boxes. Copy number variations of Chr11 are shown in purple boxes

The Tr01-Haplotype A nucleus of *T. fuciformis* Tr01 carried three minichromosomes, Chr09, Chr10, and Chr11, each less than 1 Mb in length. With an average gene density of less than 2.4 per 10 kb, each of the mini chromosomes carried less than 200 genes, a significantly lower number than on the other eight chromosomes (2.7 or more per 10 kb, *P*-value 4.6E-05 in one-way ANOVA). The proportion of repetitive sequences in the minichromosomes was more than 50.0%, and more than twice that in the other chromosomes (25.1% or less, *P*-value 1.4E-05 in one-way ANOVA). GC contents and average gene lengths of the minichromosomes were lower than those in other eight chromosomes (*P*-value 2.2E-04 and 4.7E-04 in one-way ANOVA, respectively). Due to their low gene density and high content of repetitive sequences, Chr09, Chr10, and Chr11 are regarded as accessory chromosomes. In addition, segments 1–500 kb on Chr04 (Chr04-C1) and 1–590 kb on Chr05 (Chr05-C1) contained high percentages of repetitive sequences and low densities of genes and were therefore considered accessory compartments (Fig. 3A ring (1), (2), and (3), Additional file 2: Table S5). Similarly, the Tr01-Haplotype B genome also contained three accessory chromosomes and two accessory compartments of similar sizes (Additional file 2: Table S5). Hi-C contact signals among accessory compartments were stronger than those among core compartments (Additional file 1: Fig. S3).

Genome-wide SNP calling between two haplotypes displayed a landscape of gradient heterozygosity. Tr01-Haplotype A genome was divided into 2810 bins of 10 kb size each. Sequence pairs of 21.8% of bins were nearly identical (> 99.9%), representing low heterozygosity. Bins with sequence differences of 0.1–1%, 1–2%, 2–3%, and 3–4% had similar numbers of, accounting for 15% respectively. Another 19.3% of bins had SNP densities more than 4%. Low-heterozygosity bins occurred mainly in nine regions, including four whole chromosomes (Chr04, Chr09, Chr10, and Chr11) and five compartments (Chr02:1,754,000–2,351,000, Chr03:1,862,000–2,012,000, Chr05-C1, Chr06:936,000–1,155,000, Chr07:1,006,000–1,190,000). All accessory chromosomes and compartments were located in low-heterozygosity regions (Fig. 3A ring (4)), indicating a different evolutionary path for these regions from the rest of the genome.

Analysis of the complete set of benchmarking universal single-copy orthologs (BUSCO) for Basidiomycota in the *T. fuciformis* genome was used to assess the completeness of our genome assembly and to assist in distinguishing core and accessory chromosomes. Between 96.9 and 97.2% of conserved Basidiomycota BUSCO genes could be predicted in the Tr01-Haplotype A and B genomes. Only one BUSCO gene was detected in the 22 subtelomeric regions (100 kb in size) of the Tr01-Haplotype A genome, and four were found in the 24 subtelomeric regions of Tr01-Haplotype B. An average of 0.11 genes per 100 kb in the subtelomeric sequences was much lower than the BUSCO gene density (6.1/100 kbp) in the rest of the chromosomes of both haplotypes. No BUSCO genes were detected in accessory chromosomes and compartments (Fig. 3A ring (5)), supporting the hypothesis that accessory chromosomes/compartments are non-essential.

By examining sequence read depth across the accessory regions, large-scale copy number variations were identified in about one-fifth of monospore isolates (6 of 33 strains). Loss of the entire Chr09 was detected in isolates DBZ07 and DBZ33 (Fig. 3B), indicating that the loss took place during meiosis. The sequence read depth of whole Chr11 was halved in isolate DBZ03, but increased by about half in isolates DBZ23, DBZ25, and DBZ29 (Fig. 3B). Germinated from a single basidiospore, sequenced genomes should be two or more karyotypes, each of which carried different copies of Chr11, indicating that gain or loss of whole Chr11 occurred in some cells after meiosis.

### Extra chromosomes in monospore isolates

**Fig. 4:**
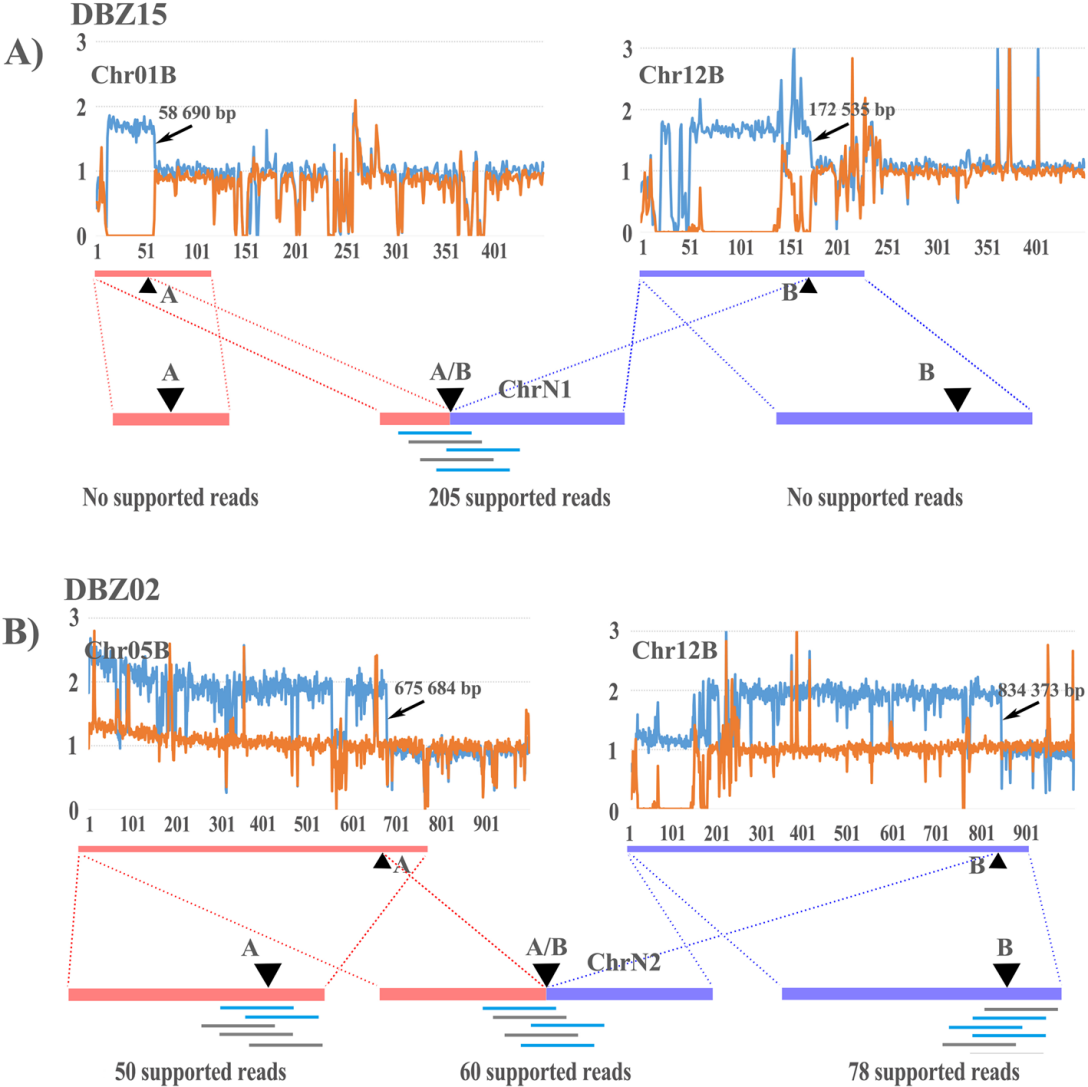
Evidence for formation of new chromosomes. ChrN1 in *Tremella fuciformis* monospore isolate DBZ15 is in panel **A**. ChrN2 in *Tremella fuciformis* monospore isolate DBZ02 is in panel **B**. **A** Above: sequence read depth of monospace isolate DBZ15 at regions Chr01B:1–450,000 and Chr12B:1–450,000 (blue traces represent DBZ15, orange traces represent DBZ43). Panel **A** Below: supported PacBio long reads that span the breakpoints at A and B in sequence read depth and the rejoining site A/B. **B** Above: sequence read depth at regions Chr05B:1–1,000,000 and Chr12B:1–1,000,000 (blue traces represent DBZ02, orange traces represent DBZ43). Panel **B** Below: supported PacBio long reads that span breakpoints at A and B in sequence read depth and the rejoining site A/B

*T. fuciformis* Tr01 Chr01B:1–58,690 (Chr01B-C1) and Chr12B:1–172,535 (Chr12B-C1) were type B genome-specific regions that are regarded as the markers for Tr01-Haplotype B architecture. In monospore isolate DBZ15, a ~1.8× sequence read depth was detected in regions of Chr01B-C1 and Chr12B-C1 (Fig. 4A). No sequence reads cross the sites of Chr01B:58,690 (site A) and Chr12B:172,535 (site B), whereas 205 sequence reads supported the linkage between Chr01B-C1 and the reverse compliment of Chr12B-C1 (Chr12B-C1 RC), suggesting that Chr01B-C1 had become linked to Chr12B-C1 RC to form a new chromosome (ChrN1). An approximate 1.8× sequence read depth also revealed two or more copies of ChrN1 in some but not all cells. In DBZ02, PacBio long reads supported the connection of upstream and downstream sequences at each of sequencing depth inflection points A and B (Fig. 4B). Furthermore, 2.0× more sequence read depth was detected in regions of Chr05B:1–675,684 and Chr12B:1–834,373 than those in DBZ43 (Fig. 4B). Sixty PacBio long reads supported the linkage between Chr05B:1–675 684 and the reverse compliment of Chr12B:1–834,373 (Chr12B:1–834,373 RC), suggesting that Chr05B:1–675,684 linked to Chr12B:1–834,373 RC to form a new chromosome (ChrN2).

ChrN1 was a minichromosome with a length of 0.23 Mb, 32.9% of which comprised repetitive sequences (Table 2), harboring 62 genes including one BUSCO and 12 repeat element-related genes. ChrN2 was a small 1.52-Mb chromosome, 16.0% of which comprised repetitive sequences, harboring 476 genes including 105 BUSCO and 72 repeat element-related genes. Due to each of component segments containing one telomere and no centromere, both new chromosomes were capped by telomeres at both ends, but did not have centromeres.

**Table 2:** Features of new chromosomes identified in monospore isolates of *Tremella fuciformis* Tr01

Chr. ID	Length (Mb)	Corresponding regions	5’ end telomere	3’ end telomere	Centromere	Repeat sequences (%)	Genes		
							Total	BUSCO	Repeat element related
ChrN1	0.23	Chr01B:1–58690 and Chr12B:1–172535 RC	+	+	−	32.9	62	1	12
ChrN2	1.52	Chr05B:1–675684 and Chr12B:1–834,373 RC	+	+	−	16.0	476	105	72

**Fig. 5:**
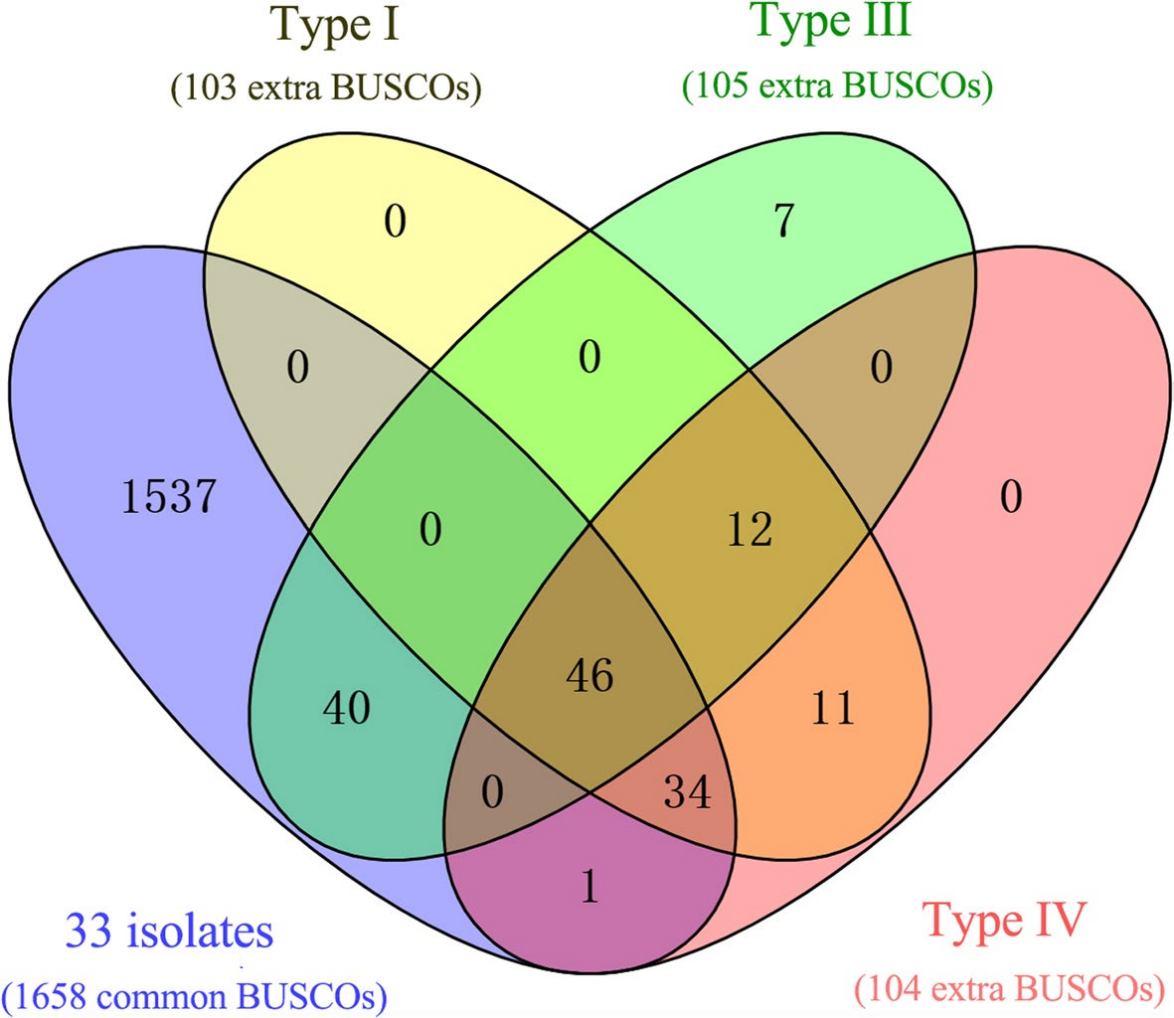
Venn diagram of BUSCOs. Yellow, green, and pink represent extra BUSCOs among Type I, III, and IV isolates, respectively. Blue represents common BUSCOs among 33 monospore isolates

Our 14 monospore isolates (42.4%) of *T. fuciformis* Tr01 were aneuploids with one or two extra chromosomes and could be clustered into four types (Table 3). Types I, II, and III included eight, one, and two isolates, contained extra Chr12B, ChrN1, and ChrN2 chromosomes, respectively, and exhibited Tr01-Haplotype A genomic architecture. Type IV was represented by three isolates, two extra chromosomes, Chr12B and ChrN1, and also exhibited Tr01-Haplotype A architecture. Thus, among 33 monospore isolates studied, 17 had Tr01-Haplotype A architecture with 11 chromosomes, 11 isolates had 12 chromosomes, and three isolates had 13 chromosomes. The genomes of 33 monospore isolates of *T. fuciformis* Tr01 contained 1658 common BUSCOs, which were necessary for the survival of each monospore isolate. Type I, III, and IV aneuploids carried ~103–105 extra BUSCOs, at least 80 of which were required for monospore isolate survival (Fig. 5).

**Table 3:** Types of aneuploidy in monospore isolates of *Tremella fuciformis* Tr01

Types	Chr. number	Extra chr.	Size of extra chr. (Mb)	Corresponding monospore isolates
Type I	12	Chr12B	1.52	DBZ18, DBZ23, DBZ25, DBZ28, DBZ29, DBZ33, DBZ46, DBZ10
Type II	12	ChrN1	0.23	DBZ15
Type III	12	ChrN2	1.52	DBZ02, DBZ08
Type IV	13	Chr12B and ChrN1	1.75	DBZ11, DBZ14, DBZ20

*Chr.* Chromosome

### Genomic biases of *T. fuciformis* Tr01 monospore isolates

One interesting feature of *T. fuciformis* Tr01 is the disorder in basidiospore formation. The four-celled phragmobasidia with cruciate septa were commonly found with fewer spores than normal as shown in the micrograph of mature Tr01 fruiting bodies (Additional file 1: Fig. S11A). The four-celled phragmobasidia of Tr01 did not generate normal number of basidiospores (Additional file 1: Fig. S11B).

**Fig. 6:**
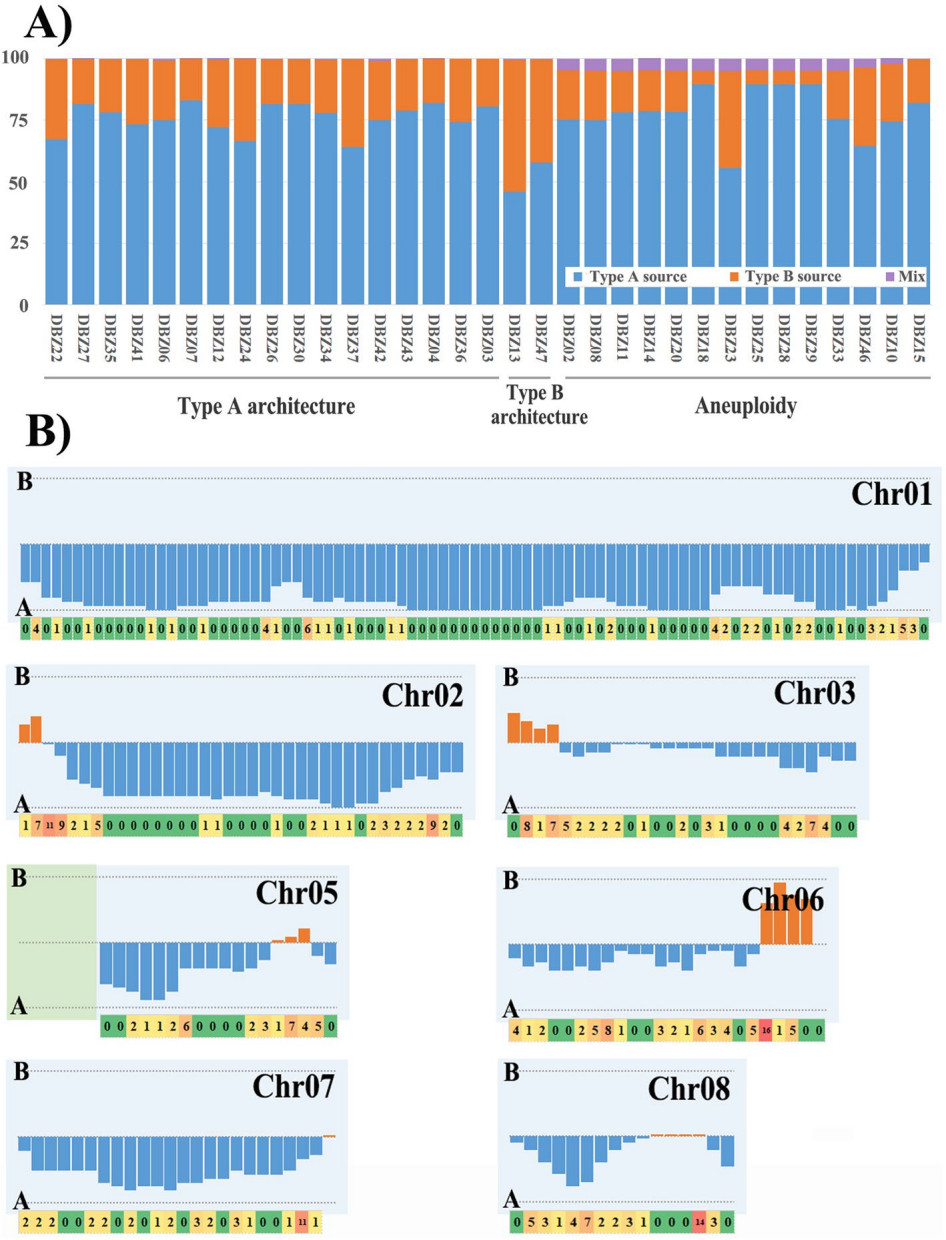
Genomic bias of monospore isolates from *Tremella fuciformis* Tr01. **A** Genomic sequence bias of each monospore isolate from *T*. *fuciformis* strain Tr01. **B** Sequence bias and homologous recombination events in each bin (100 kb/bin) of high-heterozygosity regions relative to the Tr01-Haplotype A genome. Upper part of the panel representing each chromosome: the height of blue bars represents the number of isolates from Tr01-Haplotype A source sequence (when more than half of isolates), and the height of orange bars represents the number of isolates from Tr01-Haplotype B source sequence (when more than half of isolates). Lower part of the panel representing each chromosome shows the number of homologous recombination events occurring in the corresponding bin. Colors also distinguish the relative numbers of homologous recombination events, from green for 0 though yellow, orange, and red for increasing numbers of such events

Among 33 *T. fuciformis* Tr01 single basidiospore isolates, 14 (42.4%) were aneuploids with one or two extra chromosomes. All of these aneuploidy exhibited Tr01-Haplotype A genomic architecture, whereas the extra chromosomes were derived from whole chromosomes or from recombination of chromosomes in the Tr01-Haplotype B genome. Another 17 isolates also featured Tr01-Haplotype A genomic architecture (Fig. 6A). Only isolates DBZ13 and DBZ47 (6%) exhibited Tr01-Haplotype B architecture (Fig. 6A). Genomic architectures of monospore isolates from Tr01 were predominantly homologous to the Tr01-Haplotype A nucleus.

SNP calling in the Tr01-Haplotype A genome revealed that SNP densities in 1770 out of 2810 bins (10 kb/bin) were greater than 1%. These heterozygous bins were used to characterize the origins of sequences in the genomes of monospore isolates as having arisen during meiosis. All *T. fuciformis* Tr01 monospore isolates harbored both Tr01-Haplotypes A and B nuclear sequences, indicating that they are meiotic products of Tr01 (Fig. 6A). More than half of the genomic sequences of all isolates except for DBZ13 originated from the Tr01-Haplotype A nucleus (Fig. 6A). The genomes of DBZ13 and DBZ47 featured Tr01-Haplotype B architecture and 54 and 42% of their sequences, respectively, originated from the Tr01-Haplotype B nucleus. Approximately 5% of the bins of all aneuploid isolates were heterozygous, except for DBZ15, DBZ10, and DBZ46, and the ratio of extra chromosome length corresponded to their genome size (Fig. 6A). The extra chromosome of DBZ15 was composed only of Tr01-Haplotype B-specific regions. Loss of heterozygosity took place in the extra Chr12B region from 376 to 1134 kb in DBZ10, and in the extra Chr12B regions from 387 to 699 kb and 1006 to 1289 kb in DBZ46, which led to the percentage heterozygosity of bins in these isolates to be less than their ratio of extra chromosome length to genome size.

Nuclear sequence biases and inter-nucleus genetic exchange events in *T. fuciformis* Tr01 were investigated in the highly heterozygous regions in the Tr01-Haplotype A genome (100 kb/bin, Fig. 6B). Most sequences on Chr01, Chr02, and Chr07 in most isolates originated from the Tr01-Haplotype A genome. However, the sequences of bins Chr02:1–200,000, Chr03:1–400,000, Chr05:1,900,000–2,200,000, and Chr06:1,900,000–2,300,000 were biased towards those of the Tr01-Haplotype B genome and accounted for ~5% of genome size. Less sequence bias was detected in the regions Chr03:400,000–2,670,835, Chr06:1–1,900,000, and Chr08:700000–1,584,971. A total of 368 recombination events were detected at 277 recombination sites in the highly heterozygous regions of 33 monospore isolates (Additional file 2: Table S6). Both recombination events and sites were unevenly distributed in the whole *T. fuciformis* Tr01 genome (Fig. 6B). More recombination events occurred in bins with wide differences in nuclear sequence bias to their neighbors. Four or more monospore isolates underwent genetic exchanges at each of 16 recombination sites regarded as recombinant hotspots (Additional file 2: Table S6). Interestingly, some recombination sites were very close to each other. For example, only 15 bp separated the recombination sites Chr02A:142183–142201 and Chr02A:142217–142302.

## Discussion

The dikaryotic phase dominates most of the life cycle of basidiomycete fungi. During this phase, each individual carries two different haploid nuclei until shortly before spore production. Interactions between the two nuclei, such as inter-nucleus genetic exchanges, have long been of interest, yet remain mostly unknown. Thus, the assemblies of haploid genomes for each nucleus of eukaryotic fungi have been a key resource to address these genomic processes. Advances in long-read sequencing data combined with chromosome scaffolding methods have now made chromosome-level genome assemblies of many species achievable [16–18]. Here, we used PacBio HiFi reads of Tr01 to generate two sets of contigs by HiFiasm in Hi-C mode. Each set of contigs was further assembled into T to T chromosomes separately with assistance of polished Nanopore ultra-long reads. Due to the phenomenon that chromosomes within same nucleus having stronger Hi-C contact signals than those from different nuclei, the program NuclearPhaser reassigned the chromosomes into two different nuclear genomes, Haplotype A and B genomes. Homologous chromosomes of Chr04, Chr09, Chr10, and Chr11 had remarkably high in sequence similarity (> 99.97%), but mainly differed in SVs. Most of the SVs were transposable elements, which widely distributed in the whole genome. Hi-C contact signals were unable to phase these chromosomes correctly. LTR683s were Haplotype A bias LTRs, which were also rich in Chr04A, Chr09A, Chr10A, and Chr11A. Situation was same for Haplotype B bias LTR1239s. The features of LTR683 and LTR1239 help us to phase low-heterozygosity chromosomes well. Due to being generated independently, genomic sequences of DBZ04 also verified the assembly of two nuclear genomes of Tr01. Sequence mapping of Nanopore ultra-long reads on Haplotype A and B genomes, particularly on these recombination and low-heterozygosity regions, provided additional evidence for assembled accuracy of Tr01 genomes. Our research provided two sets of haploid nuclear genomes of Tr01, each of which was gap-free, and verified by one or more sets of data.

The genomes of some fungi are highly dynamic and exhibit an architecture with two discrete types of genomic regions comprising core and accessory compartments [19, 20]. Accessory compartments can represent entire chromosomes that have become embedded into core chromosomes and are considered non-essential for fungal growth and propagation [21, 22]. The notable characteristic features that distinguish accessory compartments from core chromosomes are their high repeat contents and low gene densities. We identified three accessory chromosomes and two accessory compartments in each haplotype of *T. fuciformis* Tr01 due to their significantly higher repeat contents and lower gene densities compared to other genomic regions, the first report of such discrete genomic architectures in basidiomycetes [23]. We detected no BUSCO genes in these accessory compartments/chromosomes and found that the monospore offspring DBZ07 and DBZ33 lost the whole Chr09 during meiosis. Both of these results provided evidence supporting the hypothesis that accessory compartments/chromosomes are generally unnecessary for cell viability in some fungi, as previously reported [24]. Genes residing in accessory compartments tend to evolve faster, compared with the slow-evolving housekeeping genes that reside on core chromosomes [24]. However, we found that the corresponding sequences of accessory regions between the A and B nuclei were nearly identical, compared with the high heterozygosity of most core regions. A possible explanation for such a contradiction is that accessory compartments/chromosomes from one nucleus have been replaced by related sequences in the other, resulting in a loss of heterozygosity. Most two-speed genomes of fungi have been detected in filamentous phytopathogens [23], and their accessory compartments carry more rapidly evolving virulence-associated genes [25]. It provided evidence that the accessory chromosomes/compartments in *T. fuciformi*s might be associated with its interaction with *A. stygium*.

In eukaryotes, chromosome structure is well preserved within members of the same species. The rate at which novel chromosomes form is apparently very low and usually associated with pathological events [3]. Still, many cases of novel chromosome formation have been described in fungi and several mechanisms for these have been proposed. Isochromosomes, composed of mirror images of one arm of a chromosome, have been detected in some *Candida albicans* isolates following antifungal drug selection [2]. Isochromosomes are supposed to have arisen from recombination between inverted repeats at centromeres. In the yeast *C. glabrata*, novel chromosomes can be generated by duplication of chromosome segments containing a centromere followed by the addition of new telomeric ends [3]. In *Cryptococcus* species, multiple chromosome translocations can occur during the repair of DNA double-strand breaks (DSBs) at centromeric retrotransposons, resulting in genome karyotype shuffling, including formation of new chromosomes [4]. Genome-shuffled isolates fail to undergo successful sexual reproduction with the parental genotype. Here, we proposed a new mechanism to address formation of new chromosome in *T. fuciformis* Tr01 progenies.

**Fig. 7:**
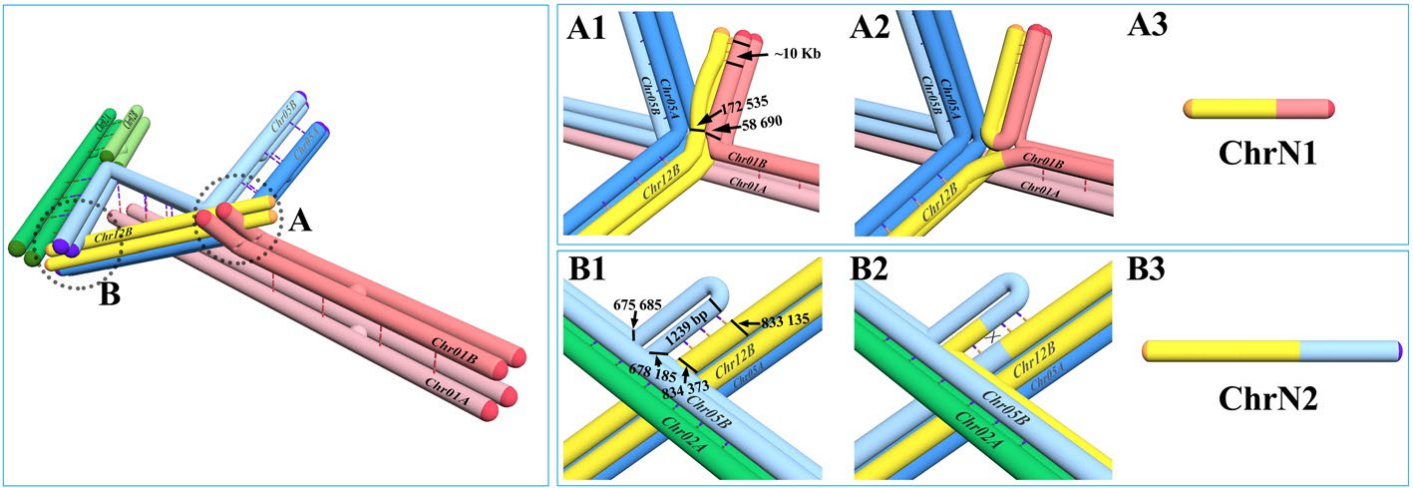
Structural model of the large complex formed during meiosis in *Tremella fuciformis* by homologous pairing. Left: structure of the large complex of seven sister chromatids. A and B are two recombination regions. Upper right: A1, A2, and A3: detailed model of recombination in region A that leads to formation of ChrN1 in monospore isolates. Lower right: B1, B2, and B3: detailed model of recombination in region B that leads to formation of ChrN2 in monospore isolates

During prophase I of meiosis, homologous maternal and paternal chromosomes or regions paired. Partial homologies among Chr01A, Chr01B, Chr02A, Chr02B, Chr05A, Chr05B, and Chr12B allowed them to pair and generate a large homologous complex (Fig. 7, left). The right arm of Chr01B paired up with right arm of Chr01A, leaving Chr01B-C1 unpaired with other regions of the Tr01-Haplotype A genome. Pairing of other homologous sequences allowed Chr01B:58690 to serve as an intersection among Chr01A, Chr01B, Chr05A, Chr05B, and Chr12B. Over excessive tension of the five chromosomes tended to result in breakage of some chromosomes at the intersection. No homologous sequences in the Tr01-Haplotype A genome paired up with the Tr01-Haplotype B-specific regions Chr01B-C1 and Chr12B-C1. The 10-kb subtelomeric regions of Chr01B-C1 and Chr12B-C1 were nearly identical, which allowed them to pair up with each other (Fig. 7 upper right A1). Breakages at the intersection of Chr01B (Chr01B:58,690) and Chr12B (Chr12B:172,535) and subsequent connection of Chr01B-C1 to Chr12B-C1 generated the new chromosome ChrN1 (Fig. 7, upper right A2 and A3). The left arm of Chr05B paired up with the left arm of Chr02A, leaving some regions unpaired, such as Ch05B:675,685–678,185. The Chr05B:675,685–678,185 region consisted of two copies of a 1242-bp repeat element that was 100% identical to the reverse compliment of Chr12B: 833,135–834,373. About half of the Chr12B: 833,135–834,373 region was unable to pair up with Chr05A. However, the sequences of Chr12B and Chr05B were fairly homologous in the regions related to Ch05B:675,685–678,185. Because they became adjacent, Chr12B:833,135–834,373 could then pair up with Ch05B:675,685–678,185 (Fig. 7 low right B1). Homologous recombination in this region could then generate the new chromosome ChrN2 (Fig. 7, lower right B2 and B3). Because these segments lack centromeres, they escaped attachment to the microtubules and could move to either daughter cell. Both of these new chromosomes were capped by telomeres at both ends, which allowed them to be stably inherited in daughter cells. Meiosis of asymmetric genomes can thus drive the formation of new chromosomes.

Generally, numerous basidiospores can be ejected from fruiting bodies, and have no bias towards either parental nucleus in genetic material. However, there are exceptions. In the case of *C. neoformans*, hybridization of two isolates with differing karyotypes (VYD135α and KN99a) lead to production of spores whose nuclear genomes are derived from only one of the two parental genomes [26]. The pseudosexual reproduction referred is supposed to stem from nuclear loss via hyphal branches. Our study revealed three peculiar features related to basidiospores of *T. fuciformis* Tr01. The mature fruiting bodies produced plenty of four-celled phragmobasidia with cruciate septa, from which few basidiospores generated. Among 33 monospore isolates studied, 31 had Tr01-Haplotype A architecture, whole genomic sequences of 32 showed a biased inheritance to the Tr01-Haplotype A genome. Besides, 14 monospore isolates (42.4%) were aneuploids with one or two extra chromosomes. Meiosis analyses of the asymmetric dikaryotic genome may provide proper explanation for these phenomena.

During prophase I of meiosis, homologous regions among *T. fuciformis* Tr01 Chr01A, Chr01B, Chr02A, Chr02B, Chr05A, Chr05B, and Chr12B paired up to form a large homologous complex, leading to some inter- and intra-chromosomal, physical contacts (Additional file 1: Fig. S12A left). These contacts facilitated abnormal rearrangements within or between these chromosomes, which might result in uneven distribution of genetic materials into daughter cells and fragmented chromosomes (Additional file 1: Fig. S12A middle). Uneven distribution of genetic materials led to one or both daughter cells lacking some core genes essential for their survival. Fragmented chromosomes were unstably inherited and are gradually lost, leading to the inviability of some daughter cells (Additional file 1: Fig. S12A right).

During metaphase I, each chromosome was able to migrate into either daughter cells by spindle fibers (Additional file 1: Fig. S12B left). However, disruption of combination of Chr01A, Chr02A, and Chr05A (Tr01-Haplotype A architecture) resulted in daughter cells lacking essential genes for survival. For example, exchange of just Chr01 occurring during metaphase I process can result in two different types of daughter cells: type A lacking a fragment of Chr01A:1-211832 (including 148 BUSCOs) and type B containing a pair of the homologous sequences (Additional file 1: Fig. S12B right). Moreover, type B daughter cells tended to lose other core genes due to free movement of Chr12B. As a result, few basidiospores are able to survive, most of which are characterized by Tr01-Haplotype A architecture.

Tr01-Haplotype B genome included a core chromosome Chr12B that lacked a centromere. Without the ability to attach to kinetochore microtubules, Chr12B was more likely to migrate to daughter cells with Tr01-Haplotype A architecture following its pairing with Chr05A during meiosis (Additional file 1: Fig. S12C left). Chr12B contained 103 BUSCOs, at least 80 of which were required for monospore isolate survival. As a result, many monospore isolates with Tr01-Haplotype A architecture contained an extra Chr12B (Additional file 1: Fig. S12C right, 33%) and few monospore isolates (6%) exhibited Tr01-Haplotype B architecture. Accordingly, isolates with Tr01-Haplotype A architecture contained more sequences deriving from Tr01-Haplotype A genome. Similarly, some monospore isolates contain an extra ChrN1 or ChrN2.

Although sporelessness can hinder the reproduction and propogation of fungi, as every coin with two sides, the sporeless trait can be of benefit to mushroom cultivation because massive production of spores can cause many serious problems, including respiratory allergic reactions to workers [27, 28] and mushroom production losses. However, sporelessness often coincides with poor quality and yield in mushrooms [29]. So far, it has been difficult to breed sporeless varieties of high quality and yield even via manipulations at the nucleotide and gene level. Now, our study provides a new approach for sporeless mushroom breeding. Homologous recombination between two genomes with different architectures might facilitate abnormal rearrangements within or between chromosomes, and then resulted in uneven distribution of genetic materials into daughter cells. Uneven distribution of genetic materials led to partial daughter cells lacking some core genes essential for their survival. Hybridization of two compatible haploids with different genome architectures would be one possible way to breed sporeless mushroom varieties.

## Conclusions

We sequenced and assembled a complete, high-quality dikaryotic genome of *Tremella fuciformis* Tr01 with two haplotype genomes. Resulting from several inter-nuclear chromosomal arrangements, two nuclear haploid genomes within Tr01 are asymmetric in chromosome number, length, and content. Each nucleus had a two-speed genome, harboring three accessory chromosomes and two accessory compartments with no BUSCO genes, easy to be lost, and can affect horizontal chromatin transfer between nuclei. Among 33 monospore isolates sequenced, 31 belong to Tr01-Haplotype A genome architecture, and their genome sequences were biased towards those of the Tr01-Haplotype A nucleus. More than one-third of monospore isolates were aneuploidy, carrying one or two extra chromosomes including Chr12B and two new chromosomes ChrN1 and ChrN2. A hypothesis was proposed that the homologous regions of seven sister chromatids paired into a large complex during meiosis, which was followed by inter-chromosome recombination at physical contact sites and formation of new chromosomes. Irregular migration during meiosis and mitosis of centromere-free chromosomes such as Chr12B, ChrN1, and ChrN2 resulted in aneuploidy of some daughter cells and inviability of most other daughter cells.

## Materials and methods

### Strains

*T. fuciformis* Tr01 is available from the Center for Mushroom Germplasm Resources Management and Preservation of Fujian Province. A total of 33 single basidiospores (monospores) were isolated from Tr01 and germinated into monokaryotic strains named as DBZ plus number. For monospore isolation, a piece of matured fruiting body was suspended in autoclaved conical flask with stopper 2 days. Basidiospores were collected by bottom washing of autoclaved water, and observed under optical microscope. After serial dilution, plate cultivation and streak plate, single colony in each plate was isolated as a monospore isolate.

### Genome sequencing

Based on our analyses, the genomes of yeast-like cells can be unstable, whereas those of mycelia appear to be relatively stable (unpublished). *T. fuciformis* Tr01 mycelia are generally found mixed with those of *A. stygium* in the spawn. Mycelial colonies of Tr01 mixed with *A. stygium* were collected for PacBio circular consensus sequencing (ccs), Oxford Nanopore sequencing, and chromosome conformation capture (Hi-C) analyses. High molecular weight DNA extraction, DNA quantification and size assessment, library construction, PacBio ccs sequencing, and Oxford Nanopore sequencing were performed at Frasergen Bioinformatics (Wuhan, China) to obtain ~100✕ high-fidelity (HiFi) PacBio and Nanopore ultra-long reads, respectively. Sample preparation using Sau3A restriction endonuclease digestion and Hi-C sequencing were also performed at Frasergen Bioinformatics. Yeast-type cells of DBZ04 were sequenced on a PacBio Sequel system to obtain continuous long reads for haplotype genome assembly, which was performed at Novogene (Beijing, China). DNA from yeast-type cells of each monospore isolate and Tr01 dikaryotic isolate was also sequenced at Novogene on an Illumina X-10 sequencing platform to obtain ~4 Gb paired-end reads.

### Genome assembly of monokaryotic isolate DBZ04

We used an iterative approach to assemble and improve the genome of the *T. fuciformis* monospore isolate DBZ04. Assemblies were generated from the PacBio sequences using Canu v. 1.7 [30] assuming a genome size of 28 Mb and minimum read length of 1000 bp, and allowing an error rate of 0.045. Telomeres were detected by manually searching for the tandem repeats TTA(G)_3–5_ or AAT(C)_3–5_ at each end of contigs and were then used to assess chromosome number. Contigs with telomeres on both ends were considered complete chromosomes. Hi-C reads were used to identify connections between contigs in a scaffold with gaps using the programs HiC-Pro 2.10.0 [31] and HiCPlotter [32]. Sequences from HGAP assembly were then used to fill these gaps. The rDNA region in fungal genomes consists of a tandem array of tens to hundreds of rDNA repeat units with near-identical sequences around 10 kb in length [33]. Contigs containing rDNA sequences on one end or the other are assumed to be linked to the rDNA region. The copy numbers of rDNA repeat units are counted as a ratio of their sequencing depth to that of single-copy genes. The positions of centromeres were estimated using Hi-C data with the method described by Varoquaux [34] as regions of high inter-chromosomal interaction due centromere-centromere contacts.

### Assembly of each nuclear genome in *T. fuciformis* Tr01

Mycelial colonies of *T. fuciformis* Tr01 mixed with some *A. stygium* were collected and used for PacBio circular consensus sequencing and Nanopore sequencing. Long high-fidelity (HiFi) reads and Nanopore ultra-long reads were classified as either *T. fuciformis* Tr01-related or *A. stygium-*related, by performing blastn against the DBZ04 genome and the *A. stygium* MG137 assembly (accession: GCA_003049155.1), respectively. Tr01-related HiFi reads were generated into two sets of contigs by HiFiasm 0.15.3-r339 [35] with Hi-C mode. Each set of contigs was further assembled separately. Complete chromosomes and chromosome number were assessed by telomeres. rDNA region-related contigs were filtered out, and assembled into a chromosome with the region. Mitochondria-related contigs were assembled into a circular DNA separately. Tr01-related Nanopore reads were corrected by Ratatosk 0.7.6 [36] using its Illumina sequencing reads. Polished ultra-long reads (> 100 kb) were used to link the rest contigs into T to T chromosomes. Program NuclearPhaser reassigned all the chromosomes into two different haplotype genomes. Genomic assemblies of Tr01 were further verified by comparison with DBZ04 genome, mapping of Nanopore ultra-long reads, and analyses of Hi-C data.

### Genome annotation

Annotations of the protein-coding genes encoded in each nuclear genome of *T. fuciformis* Tr01 were based on transcript evidence, ab initio gene predictions, and homologous protein evidence, all of which were implemented in the MAKER pipeline [37]. RNA sequencing was performed for the samples of yeast-type cells grown in PDB at logarithmic phase, mycelial type cells grown in PDA at 21 ℃ for 20 days, colloid-type cells grown in PDA at 21 ℃ for more than 1 month, mycelial colonies (mixed with *A. stygium*) in sawdust medium, and fruiting bodies with a diameter of 8 cm.

These RNA-seq datasets were merged and then assembled using Trinity V2.2.0 [38] with a genome-guided assembly strategy using default parameters. Assembled transcripts were selected using the PASA program [39] for further assembly into nearly full-length transcripts, which were then imported to data training programs including SNAP [40], GENEMARK [41], and AUGUSTUS [42]. Homologous protein sequences from *Kwoniella mangrovensis*, *Cryptococcus neoformans* var. *grubii* strain H99, *Cryptococcus gattii* strain WM276, and *Tremella mesenterica* DSM 1558 were obtained for genome annotation. Assembled transcripts, gene models from training programs, and homologous protein sequences were imported into the MAKER pipeline to generate a comprehensive set of putative protein-coding genes.

Benchmarking universal single-copy orthologs (BUSCO) analysis can be used to assess the completeness of genome assembly and identify accessory regions of genomes. BUSCOs were predicted using the software BUSCO v5.2.2 [43] with the basidiomycota_odb10 dataset as the genome mode setting. BUSCOs should not be detected in accessory chromosomes and compartments.

### Repetitive sequence annotation

The whole *T. fuciformis* genome assembly was used to detected full-length LTR retrotransposons using LTRharvest [44]. Sequences obtained were filtered using following parameters: > 400 bp, > 5 copies per genome (blastn, E-value = 10–15); or one or more significant hits to described LTR retrotransposon in the repbase peptide database (blasts, E-value = 10 − 5). Filtered results were merged and clustered at 80% similarity using USEARCH [45] to create the species-specific library, in which repetitive sequences were annotated using program RepeatMasker [46].

### Copy number estimation of ITS repeat unit

Some contigs in the *T. fuciformis* genome contained tandem arrays containing both ITS repeat units and sequences encoding ribosomal rRNAs: 18S rDNA, ITS1, 5.8S rDNA, ITS2, and 28S rDNA. ITS repeat units were identified by performing blastn queries with these sequences against the contigs. A 500-bp fragment was extracted from every 2000 bp in ITS repeat unit, and used as a query for blastn against PacBio reads to estimate sequencing coverage. The copy number of the ITS repeat unit was estimated as the ratio of sequencing coverage of a 500-bp fragment relative to single repeat fragments in other regions of the genome.

### Single-nucleotide polymorphism (SNP) and structural variant calling

Pbmm2 (https://github.com/PacificBiosciences/pbmm2) was used to align the long Hifi reads of Tr01 to its type A genome. The aligned BAM files were used to discover structural variants between the two nuclear genomes using pbsv (https://github.com/PacificBiosciences/pbsv).

Raw Illumina reads of single basidiospore isolates were filtered to remove adapters and low-quality bases (Q < 30). Filtered reads were aligned to the type A genome using the Burrows-Wheeler aligner [47] with default parameters. The output BAM file data were used to call SNPs using the Genome Analysis Toolkit GATK V3.5 [48] following the best practices workflow. Based on the aligned data, sequencing read depth was calculated with the command samtools depth [49]. SNP density was calculated by the command vcftools SNPdensity [50].

### A or B haplotype-specific genes and chromosomal synteny analyses

Predicted genes in the type A or B genomes were each mapped to the other genome using blastn. The type A or B haplotype-specific genes were defined as having no blast hits in the other genome. Haplotype sequences of the type A or B chromosome were aligned using MUMmer4.x [51] with nucmer-maxmatch and other parameters set as default. The regions that did not align with the corresponding genome (> 50 kb) were defined as orphan regions.

### Identification of mating type loci in *T. fuciformis*

In the Tremellaceae, *SXI1* and *SXI2* are two key genes located in the HD locus, whereas the PR locus minimally contains the *STE3*, *STE12*, *MFA1*/*2*, *CNB00600*, and *CNG04540* genes [15]. The proteins corresponding to these genes in *Tremella mesenterica* strain DSM 1558 were used to discover the HD and PR loci by blastx searches against the type A and B genomes, respectively, in *T. fuciformis*. For each of the genes in the HD and PR loci, sequence comparisons between the two haplotypes were performed by blastn of one haplotype against the other to detect polymorphisms between them.

### Supplementary Information

**Additional file 1: Fig. S1.** Genomic structure of the monospore isolate DBZ04. **Fig. S2.** Structure of rDNA region in DBZ04 genome. **Fig. S3.** Genome-wide Hi-C contact map at 20 Kb resolution derived from dikaryotic cells of Tr01. **Fig. S4.** Genome assembly of Tr01 was supported by Hi-C analyses. **Fig. S5.** Verification of structural variations by Hi-C data with 20 Kb resolution. **Fig. S6.** Assembly verification of Tr01 dikaryotic genome at telomere view. **Fig. S7.** Sequence source distribution of DBZ04. **Fig. S8.** SNP density (1 Kb window) between dikaryotic genomes of Tr01 relative to haplotype A genome. **Fig. S9.** Nuclear bias of LTR683 and LTR1239 in Tr01. **Fig. S10.** Probable tetrapolar system of Tr01 with each of mating type loci being biallelic. **Fig. S11.** Spore-less trait at morphological evidence. A) Micrograph of mature fruiting body of Tr01. B) Possible biological processes deduced by morphological evidence. **Fig. S12.** Possible meiosis activities derived from asymmetric dikaryotic genome of *T. fuciformis* Tr01. A) Large homologous complex leading to formation of fragmented chromosomes and lacking core genes of daughter cells. B) Disruption of Tr01-Haplotype A or B architecture during metaphase I resulting in lacking core genes of daughter cells. C) Possible meiosis activities of centromere-free chromosome.

**Additional file 2: Table S1.** Location and features of each centromere in the genome *T. fuciformis* DBZ04. **Table S2.** TE InDels detected in Nanopore ultra-long reads from mapping to Haplotypes A and B genomes. **Table S3.** Homologous recombination breakpoints of DBZ04 verified by mapping of Nanopore ultra-long reads. **Table S4.** Comparison of homologous chromosomes of low heterozygosity. **Table S5.** Information of two-speed genomes in two nuclei of Tr01. **Table S6.** Recombination information in each chromosome corresponding to Haplotype A genome.

**Additional file 3.** Review history.

## Data Availability

The genome assemblies generated in this study are available in the NCBI under BioProject IDs of PRJNA1023036 [52], PRJNA1023037 [53], and PRJNA1023070 [54]. The PacBio, Nanopore, Illumina, and Hi-C data used for genome assembly, as well as Illumina sequencing data of 33 monospore isolates have been deposited in the NCBI with the accession number PRJNA788978 [55]. No custom scripts and software was used other than those mentioned in the “Materials and methods” section.
