# Supplementary material for: Meiosis in an asymmetric dikaryotic genome of Tremella fuciformis Tr01 facilitates new chromosome formation
Source: Genome Biol. 2023 Dec 5;24:280. doi: 10.1186/s13059-023-03093-7 (PMC10696834; doi:10.1186/s13059-023-03093-7)
Supplement: Supplementary file 1 — Additional file 1: Fig. S1. Genomic structure of the monospore isolate DBZ04. Fig. S2. Structure of rDNA region in DBZ04 genome. Fig. S3. Genome-wide Hi-C contact map at 20 Kb resolution derived from dikaryotic cells of Tr01. Fig. S4. Genome assembly of Tr01 was supported by Hi-C analyses. Fig. S5. Verification of structural variations by Hi-C data with 20 Kb resolution. Fig. S6. Assembly verification of Tr01 dikaryotic genome at telomere view. Fig. S7. Sequence source distribution of DBZ04. Fig. S8. SNP density (1 Kb window) between dikaryotic genomes of Tr01 relative to haplotype A genome. Fig. S9. Nuclear bias of LTR683 and LTR1239 in Tr01. Fig. S10. Probable tetrapolar system of Tr01 with each of mating type loci being biallelic. Fig. S11. Spore-less trait at morphological evidence. A) Micrograph of mature fruiting body of Tr01. B) Possible biological processes deduced by morphological evidence. Fig. S12. Possible meiosis activities derived from asymmetric dikaryotic genome of T. fuciformis Tr01. A) Large homologous complex leading to formation of fragmented chromosomes and lacking core genes of daughter cells. B) Disruption of Tr01-Haplotype A or B architecture during metaphase I resulting in lacking core genes of daughter cells. C) Possible meiosis activities of centromere-free chromosome. [file 13059_2023_3093_MOESM1_ESM.docx]

**Supplementary Materials for**

**Meiosis in an asymmetric dikaryotic genome of *Tremella fuciformis* Tr01 facilitates new chromosome formation**

Youjin Deng^a,b,e^, Lin Guo^a,b,e^, Longji Lin^a,b,e^, Yuefeng Li^a^, Jinxiang Zhang^a^, Yue Zhang^a^, Bin Yuan^c^, Lina Ke^c^, Baogui Xie^a*^, Ray Ming^b, d*^

^a^ College of Life Science, Fujian Agriculture and Forestry University, Fuzhou, Fujian 350002, China.

^b^ Center for Genomics, Fujian Agriculture and Forestry University, Fuzhou, Fujian 350002, China.

^c^ Zhangzhou Institute of Agricultural Science, Zhangzhou, Fujian 363005, China.

^d^ Department of Plant Biology, University of Illinois at Urbana-Champaign, 1201 W. Gregory Drive, Urbana, IL, 61801, USA.

^e^ These authors contributed equally: Youjin Deng, Lin Guo, Longji Lin.

*e-mail: Baogui Xie, mrcfafu@163.com; Ray Ming, [rayming@illinois.edu](mailto:rayming@illinois.edu)


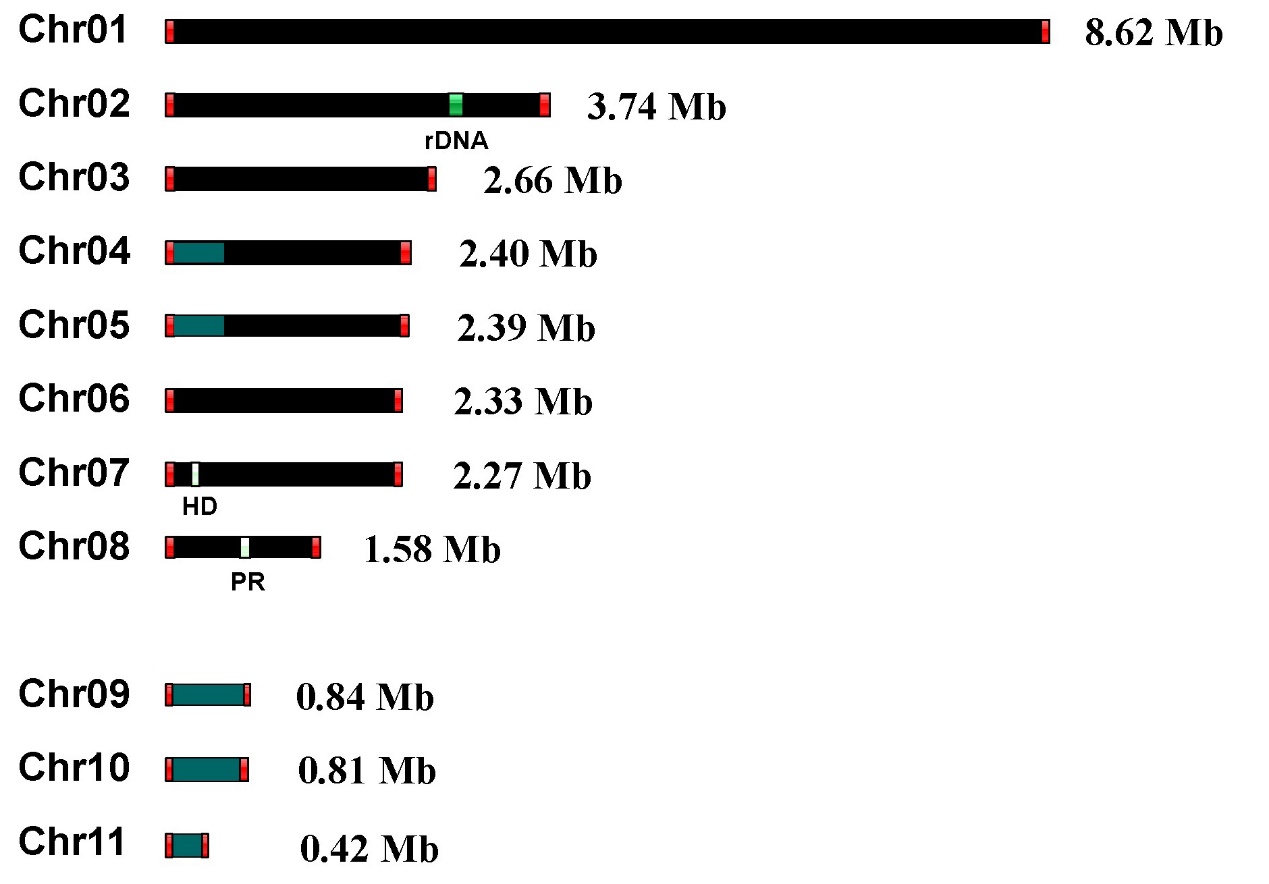


Fig. S1. Genomic structure of the monospore isolate DBZ04. The genome consists of eight core regions (black) and five accessory regons (green). Red bars represent telomeres; bright green bar represents rDNA region; white bars represent mating-type loci.


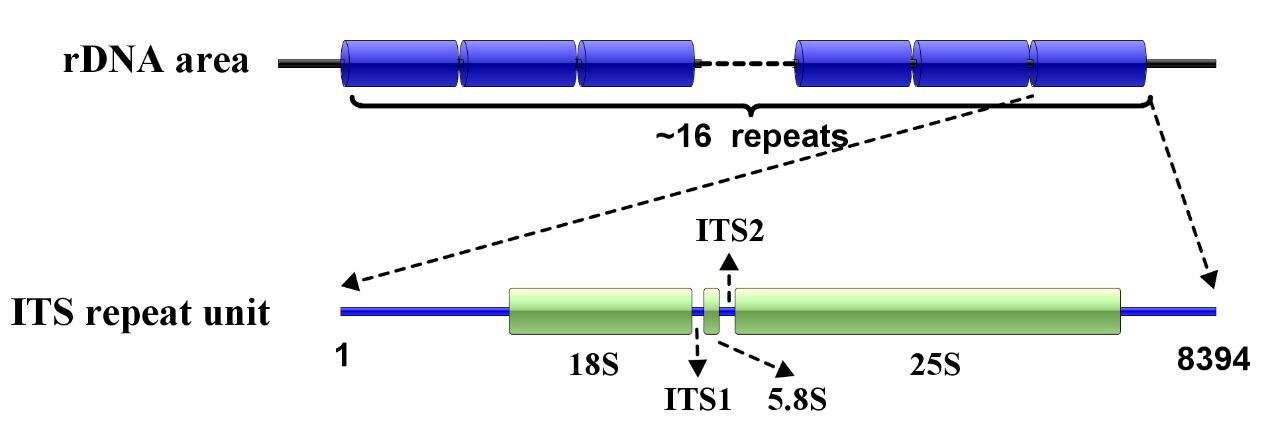
Fig. S2. Structure of rDNA region in DBZ04 genome. rDNA region consists of tandem repeat of many ITS repeat unit (dark blue bar), in which there are three rDNA genes (5.8S, 5.8S and 28 S) and two spacer regions (ITS1 and ITS2).


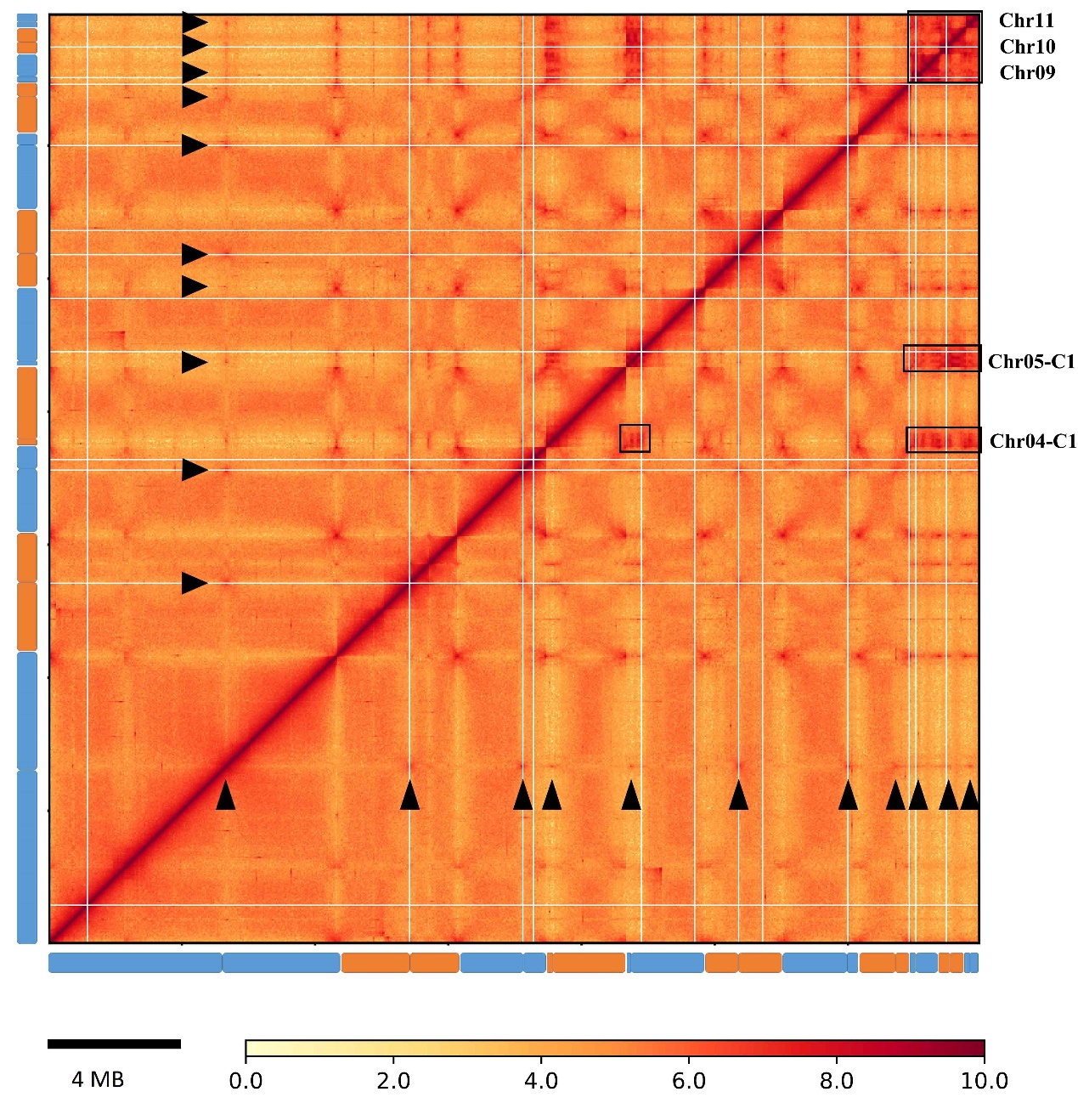


Fig. S3. Genome-wide Hi-C contact map at 20 Kb resolution derived from dikaryotic cells of Tr01. The positions of chromosomes in the whole genome data are indicated by bars along the left and bottom edges, with adjacent chromosomes indicted by different colors. Black arrowheads mark the putative centromeres. Black boxes wrap stronger contact signals among accessory compartments.


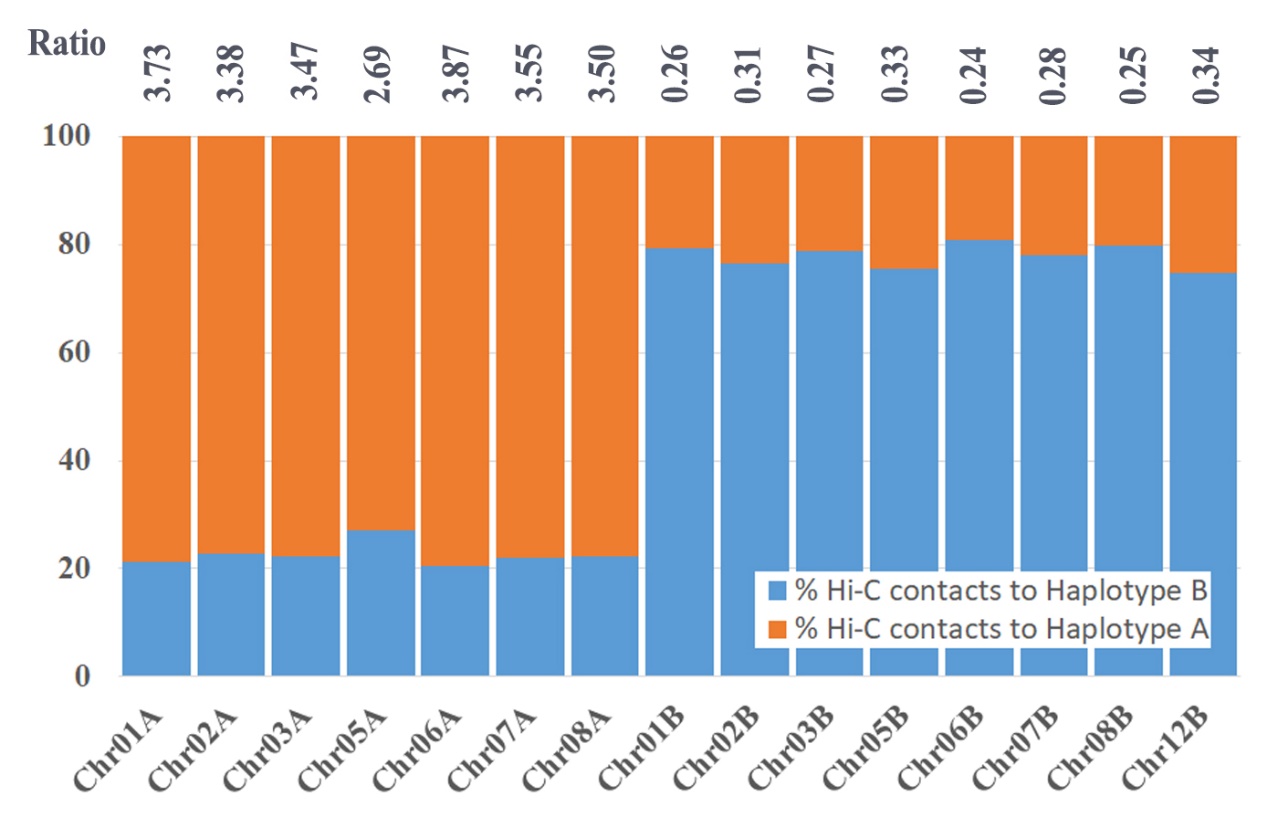


Fig. S4. Genome assembly of Tr01 was supported by Hi-C analyses. Y-axis, percentage of Hi-C contacts to Haplotype A (dark orange) and B (dark blue) of each assembled chromosome. Numbers on top line represent Hi-C contacts ratio of Haplotype A-B of each chromosome. Each pair of Chr04, Chr09, Chr10 and Chr11 being identical except for some SVs, leading to their Hi-C contacts being irregular. Their data were not included in the Figure.


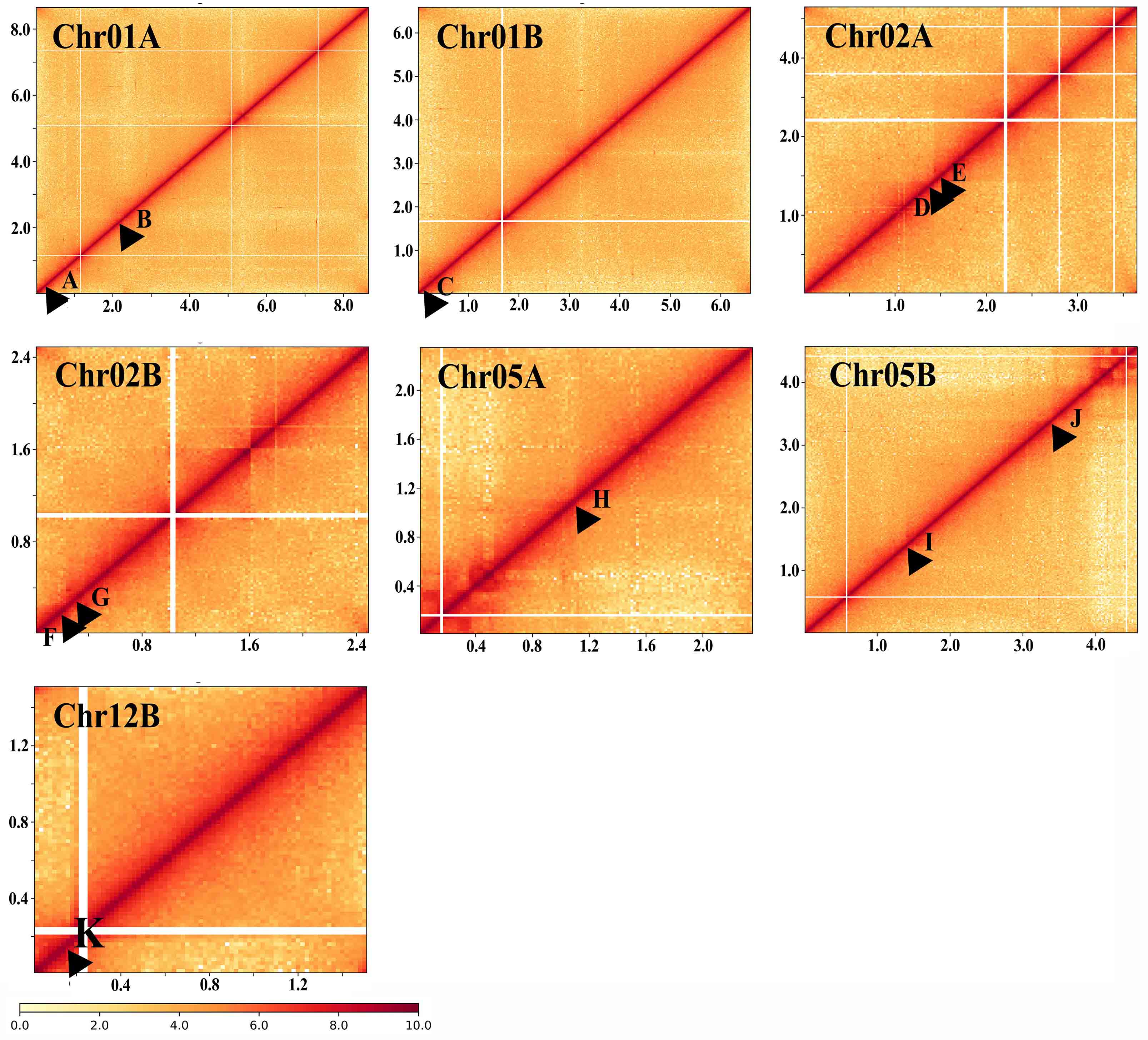


Fig. S5. Verification of structural variations by Hi-C data with 20 Kb resolution. Black arrowheads mark breakpoints of chromosome rearrangement between/within nuclear genomes.


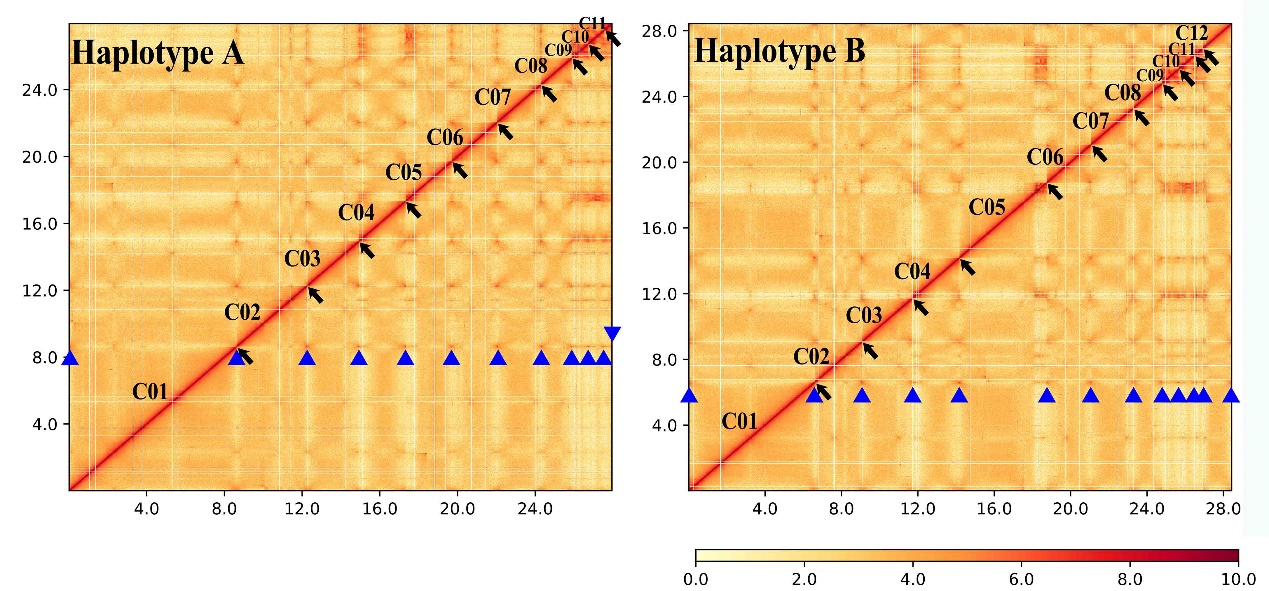


Fig. S6. Assembly verification of Tr01 dikaryotic genome at telomere view. Genome-wide Hi-C contact map of Tr01-Haplotype A (left) and Tr01-Haplotype B (right) revealed high inter-chromosomal interaction of telomeres. Black arrowheads point to boundaries among chromosomes (C01 to C11/C12). Blue arrowheads mark telomere-to-telomere contacts.


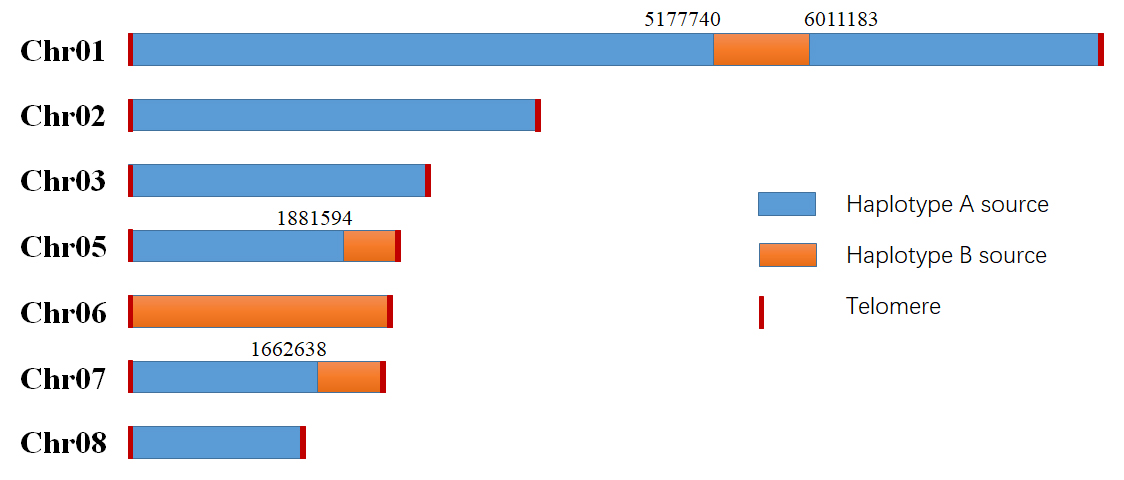


Fig. S7. Sequence source distribution of DBZ04. Information of Chr04, Chr09, Chr10 and Chr11 was not included duo to near identical sequences between two nuclear genome of Tr01 leading to undetectable recombination events in the regions.


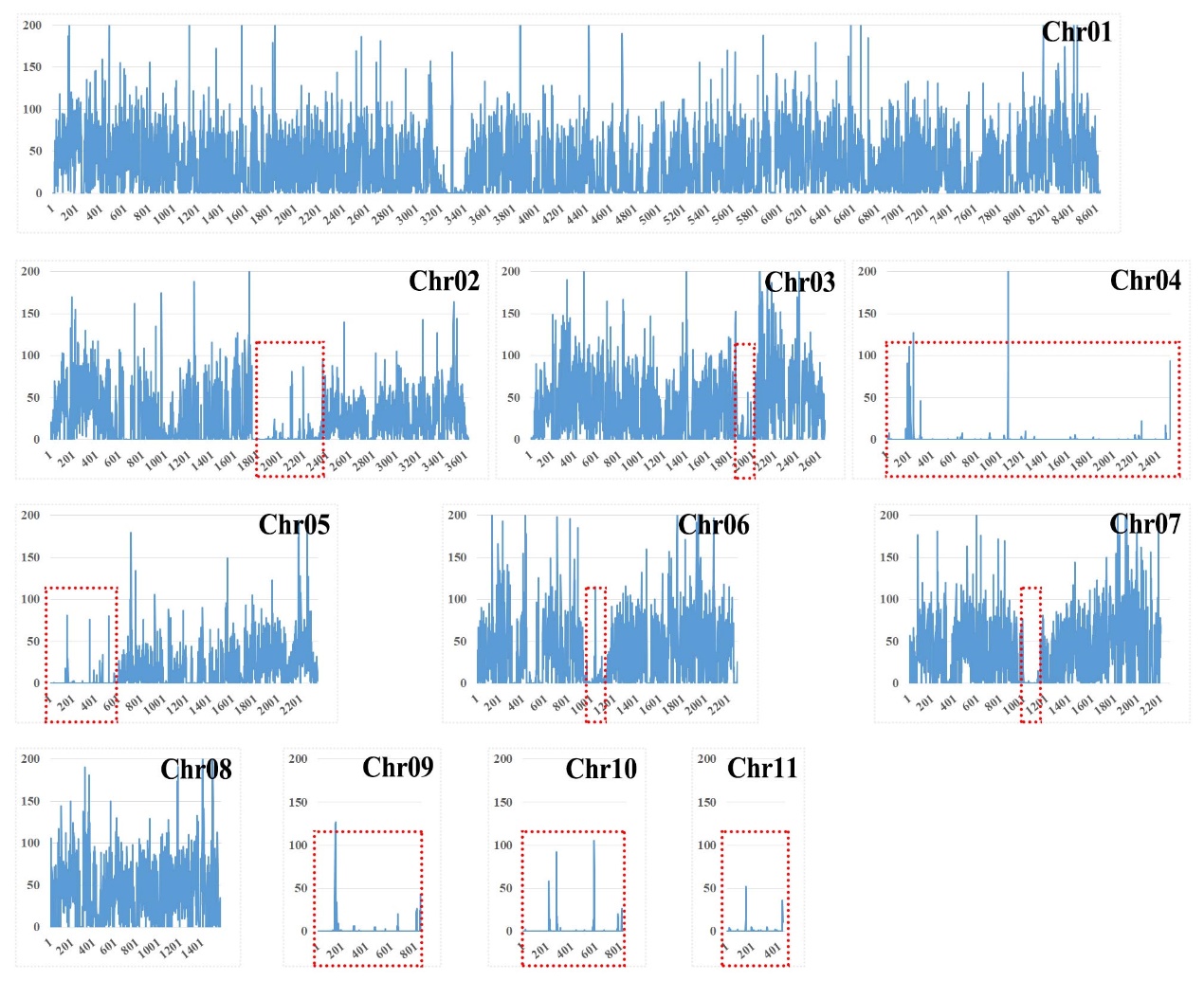


Fig. S8. SNP density (1 Kb window) between dikaryotic genomes of Tr01 relative to haplotype A genome. Regions with low heterozygosity are located in red dottled boxes.


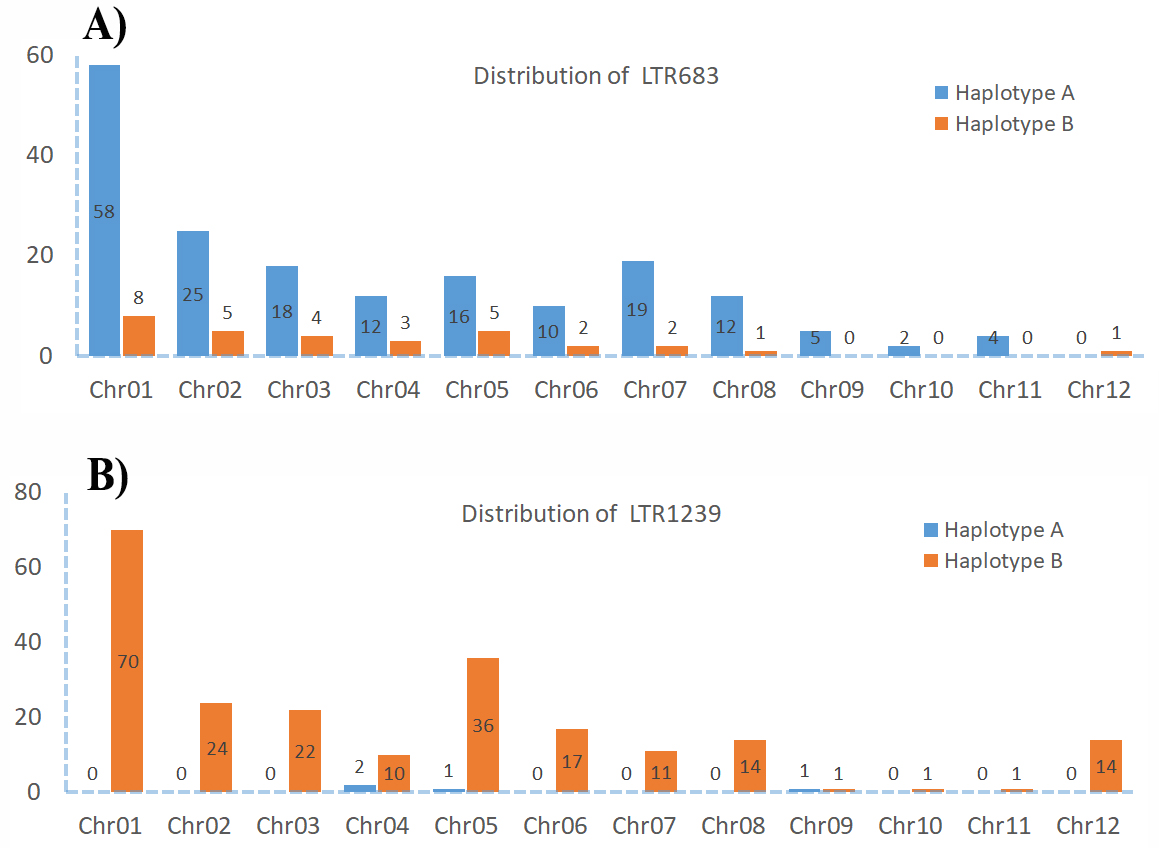


Fig. S9. Nuclear bias of LTR683 and LTR1239 in Tr01. Y-axis, number of LTRs found in the corresponding chromosomes.


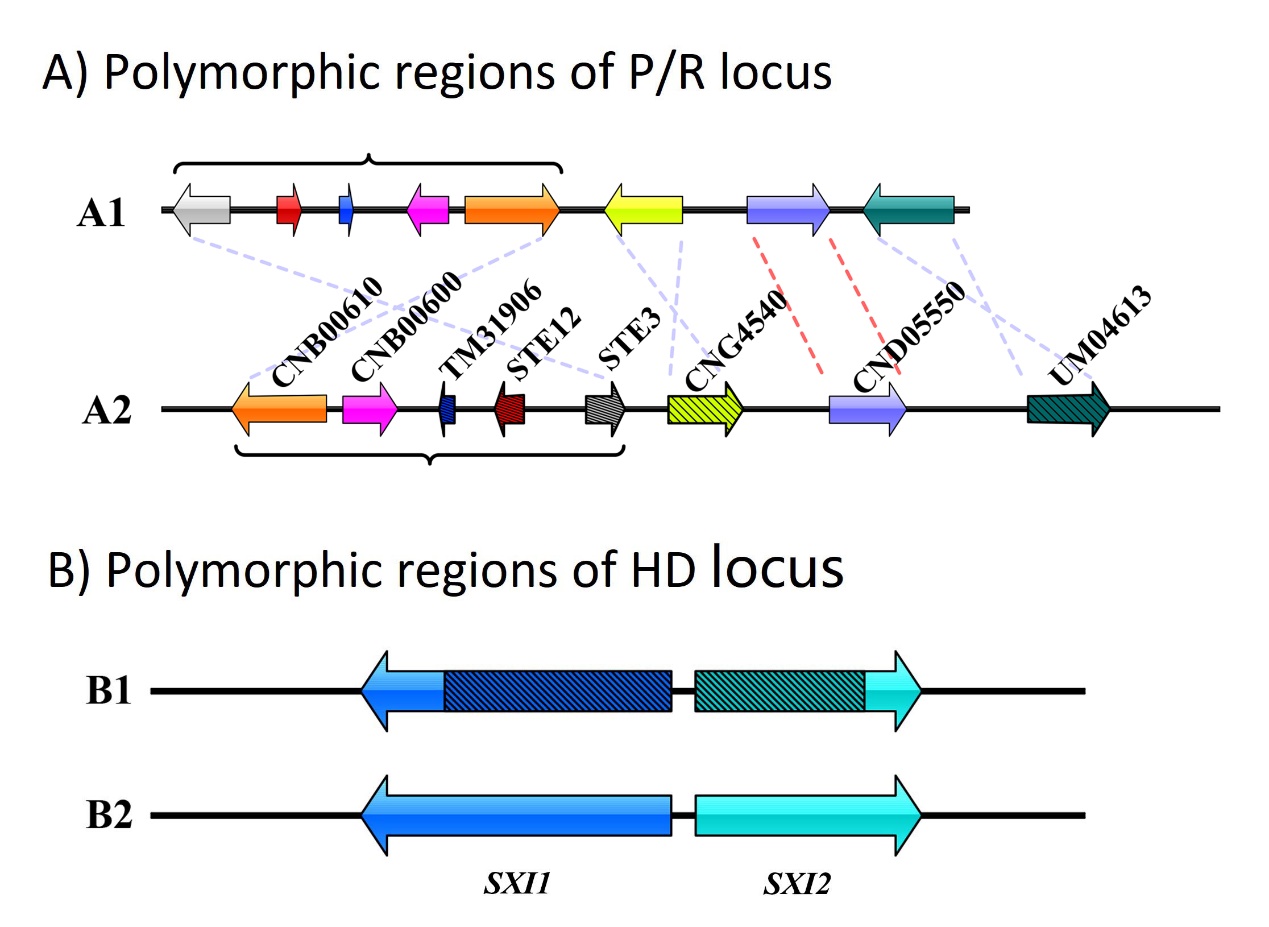


Fig. S10. Probable tetrapolar system of Tr01 with each of mating type loci being biallelic. The pheromone/receptor (*P/R*) locus contains at least the *STE3*, *STE12*, *MFA1/2*, *CNB00600*, and *CNG04540* genes, whereas homeodomain (*HD*) locus includes at least the *SXI1* and *SXI2* genes. Each kind of colored arrow represents a mating type gene. Shadow inside arrow means polymorphic regions.


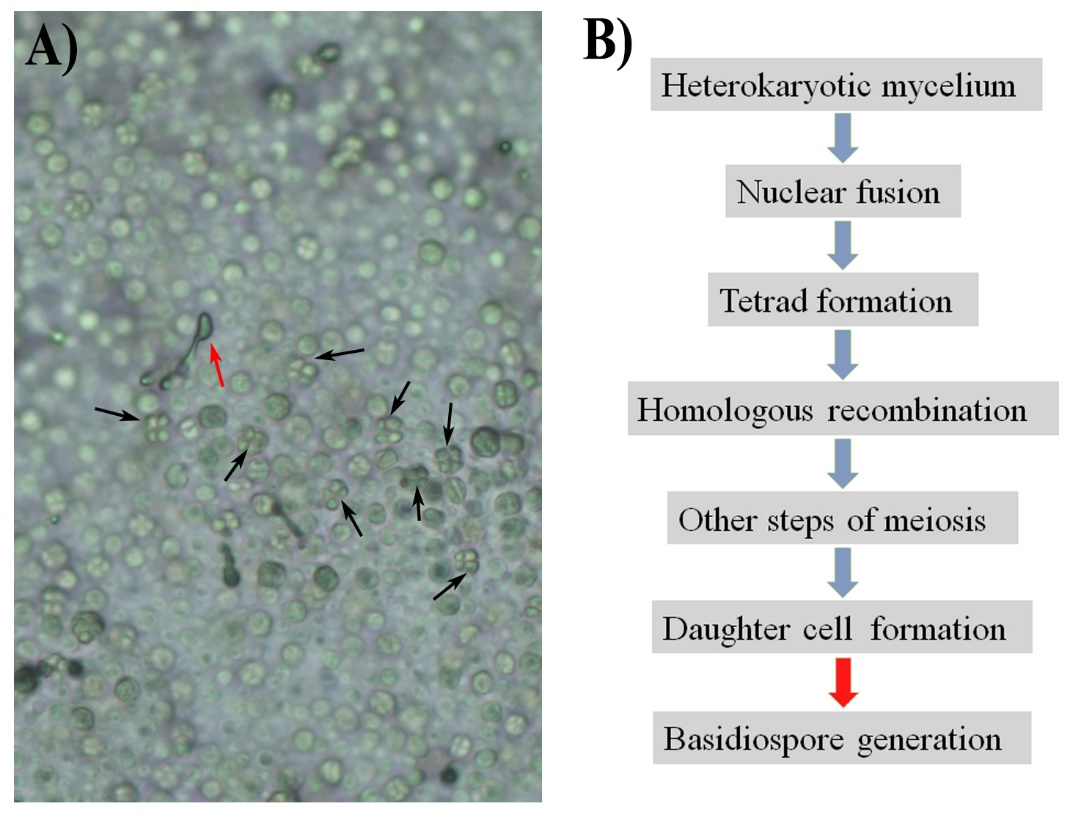


Fig. S11. Spore-less trait at morphological evidence. A) Micrograph of mature fruiting body of Tr01. Break arrows point to four-celled phragmobasidia; read arrow points to basidiospore. B) Possible biological processes deduced by morphological evidence. Blue arrows mean next step is available; read arrow means next step is unavailable.


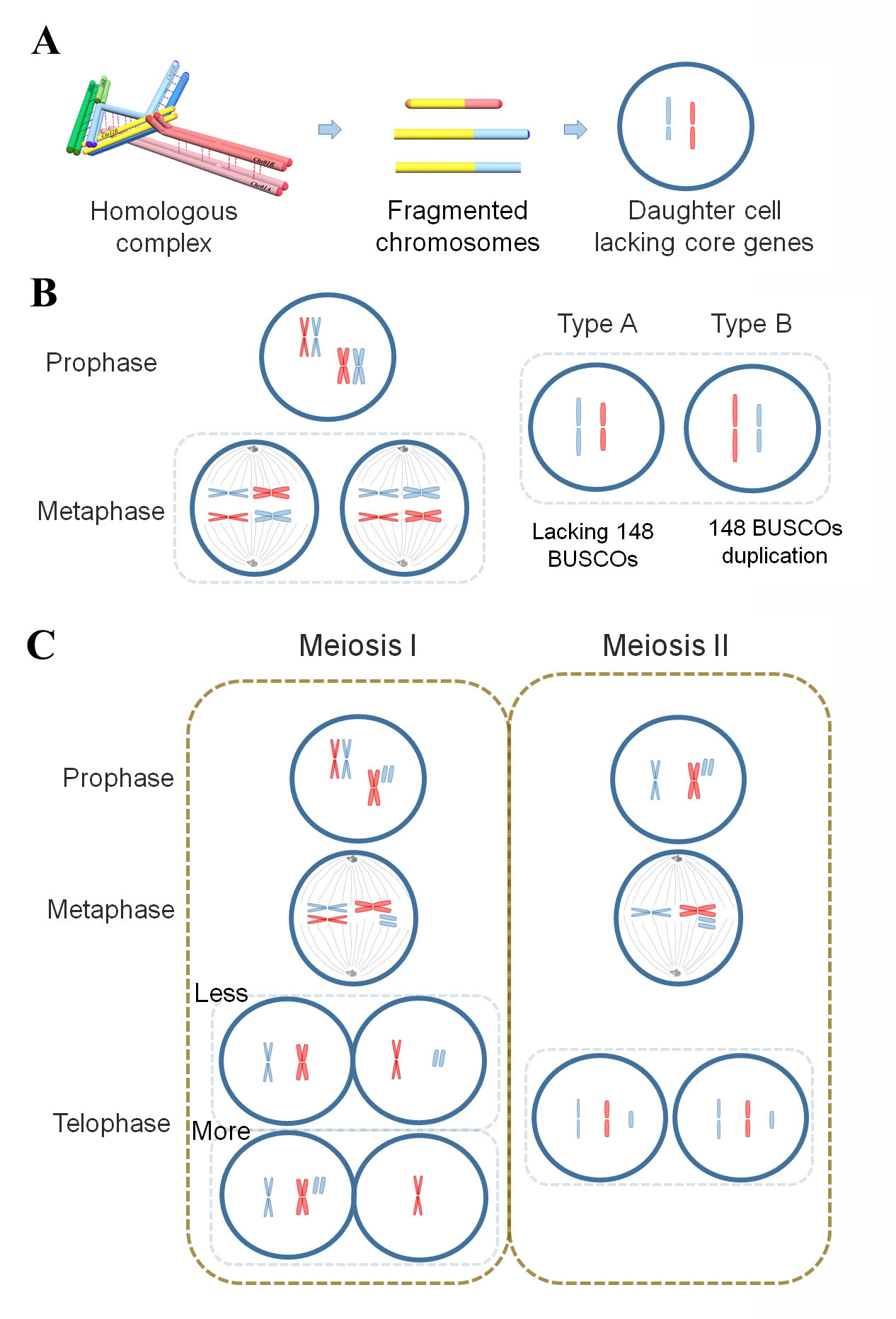


Fig. S12. Possible meiosis activities derived from asymmetric dikaryotic genome of *T. fuciformis* Tr01. A) Large homologous complex leading to formation of fragmented chromosomes and lacking core genes of daughter cells. B) Disruption of Tr01-Haplotype A or B architecture during metaphase I resulting in lacking core genes of daughter cells. Left, random migration model of chromosomes during metaphase I. Right, two different types of daughter cells resulted from exchange of Chr01 during metaphase I. C) Possible meiosis activities of centromere-free chromosome.
