# Supplementary material for: Meiosis in an asymmetric dikaryotic genome of Tremella fuciformis Tr01 facilitates new chromosome formation
Source: Genome Biol. 2023 Dec 5;24:280. doi: 10.1186/s13059-023-03093-7 (PMC10696834; doi:10.1186/s13059-023-03093-7)
Supplement: Supplementary file 2 — Additional file 2: Table S1. Location and features of each centromere in the genome T. fuciformis DBZ04. Table S2. TE InDels detected in Nanopore ultra-long reads from mapping to Haplotypes A and B genomes. Table S3. Homologous recombination breakpoints of DBZ04 verified by mapping of Nanopore ultra-long reads. Table S4. Comparison of homologous chromosomes of low heterozygosity. Table S5. Information of two-speed genomes in two nuclei of Tr01. Table S6. Recombination information in each chromosome corresponding to Haplotype A genome. [file 13059_2023_3093_MOESM2_ESM.docx]

Table S1. Location and features of each centromere in the genome *T. fuciformis* DBZ04

| Chromosome | Location  (kb) | Percentage of repeat element | Tcn1 and remnants | | Tcn6 and remnants | | Rnd-4 family-1813 and remnants | |
| --- | --- | --- | --- | --- | --- | --- | --- | --- |
|  |  |  | Number | Percentage (%) | Number | Percentage (%) | Number | Percentage (%) |
| Chr01 | 5302-5342 | 99.2 | 0 | 0 | 9 | 36 | 8 | 41 |
| Chr02 | 2182-2222 | 100 | 2 | 3 | 12 | 83 | 3 | 3 |
| Chr03 | 1921-1961 | 99.5 | 5 | 18 | 8 | 43 | 3 | 14 |
| Chr04 | 140-180 | 99.7 | 6 | 20 | 8 | 38 | 7 | 16 |
| Chr05 | 125-165 | 98.3 | 4 | 3 | 12 | 48 | 9 | 29 |
| Chr06 | 994-1034 | 99.9 | 1 | 1 | 12 | 76 | 1 | 6 |
| Chr07 | 1930-1970 | 99.2 | 1 | 1 | 9 | 32 | 4 | 13 |
| Chr08 | 1096-1136 | 69.5 | 3 | 2 | 14 | 47 | 3 | 1 |
| Chr09 | 113-153 | 99.9 | 1 | 1 | 14 | 62 | 6 | 36 |
| Chr10 | 317-357 | 76.1 | 0 | 0 | 0 | 0 | 0 | 0 |
| Chr11 | 115-155 | 73.1 | 8 | 21 | 2 | 20 | 0 | 0 |

Table S2. TE InDels detected in Nanopore ultra-long reads from mapping to Haplotypes A and B genomes

| Chromosome | TE insertions | TE deletions | Chromosome | TE insertions | TE deletions |
| --- | --- | --- | --- | --- | --- |
| Chr01A | 2 | 4 | Chr01B | 1 | 2 |
| Chr02A | 0 | 1 | Chr02B | 0 | 1 |
| Chr03A | 1 | 2 | Chr03B | 0 | 3 |
| Chr04A | 0 | 5 | Chr04B | 1 | 4 |
| Chr05A | 2 | 3 | Chr05B | 2 | 3 |
| Chr06A | 1 | 0 | Chr06B | 0 | 1 |
| Chr07A | 1 | 3 | Chr07B | 1 | 2 |
| Chr08A | 0 | 2 | Chr08B | 0 | 1 |
| Chr09A | 0 | 1 | Chr09B | 0 | 0 |
| Chr10A | 0 | 3 | Chr10B | 0 | 0 |
| Chr11A | 0 | 0 | Chr11B | 0 | 0 |
|  |  |  | Chr12B | 0 | 1 |
| Total | 7 | 24 | Total | 5 | 18 |

Table S3. Homologous recombination breakpoints of DBZ04 verified by mapping of Nanopore ultra-long reads. Read number, number of reads mapped both on upstream and downstream sequences (>10 kb) of recombination breakpoint. Upstream and downstream, upstream and downstream sequence of recombination breakpoint.

| ID | Recombination breakpoint | Haplotype A source | | | | | Haplotype B source | | | | |
| --- | --- | --- | --- | --- | --- | --- | --- | --- | --- | --- | --- |
|  |  | Read number | Similarity | | | | Read number | Similarity | | | |
|  |  |  | Upstream | Downstream | Difference | P-value |  | Upstream | Downstream | Difference | P-value |
| 1 | Chr01:5177740 | 52 | 100 | 98.7 | 1.3 | 3.54E-61 | 36 | 97 | 99.7 | 2.7 | 1.47E-45 |
| 2 | Chr01:6011183 | 45 | 96.1 | 99.9 | 3.8 | 9.40E-73 | 58 | 99.8 | 96.6 | 3.2 | 5.65E-39 |
| 3 | Chr05:1881594 | 38 | 99.8 | 96.3 | 3.5 | 1.36E-58 | 36 | 96.1 | 99.7 | 3.6 | 1.85E-43 |
| 4 | Chr07:1662638 | 45 | 99.8 | 93.1 | 6.7 | 2.08E-83 | 45 | 94.2 | 99.9 | 5.7 | 1.10E-60 |

Table S4. Comparison of [homologous chromosome](javascript:;)s of low heterozygosity.

| Chromosome | Length (bp) | Sequence similarity(%) | SNPs | Short  InDels | SV | | |
| --- | --- | --- | --- | --- | --- | --- | --- |
|  |  |  |  |  | Number | Length | Density (/100 Kb) |
| Chr04A | 2494003 | 99.99 | 122 | 21 | 51 | 215154 | 1.04 |
| Chr04B | 2405275 |  |  |  |  |  |  |
| Chr09A | 862028 | 100 | 3 | 7 | 5 | 9132 | 0.29 |
| Chr09B | 852902 |  |  |  |  |  |  |
| Chr10A | 827785 | 100 | 2 | 2 | 9 | 20255 | 0.55 |
| Chr10B | 810068 |  |  |  |  |  |  |
| Chr11A | 426448 | 99.97 | 15 | 55 | 7 | 37288 | 0.82 |
| Chr11B | 427889 |  |  |  |  |  |  |

Table S5. Information of two-speed genomes in two nuclei of Tr01

|  |  | Length | Gene number | Gene density (per 10 Kb) | Repeat content (%) | GC content (%) | Gene length (bp) |
| --- | --- | --- | --- | --- | --- | --- | --- |
| Core | Chr01A | 8654596 | 2858 | 3.3 | 11.4 | 56.7 | 2370.1 |
|  | Chr02A | 3633012 | 1095 | 3.0 | 16.5 | 56.9 | 2424.7 |
|  | Chr03A | 2670835 | 823 | 3.1 | 15.1 | 56.6 | 2326.2 |
|  | Chr04A-C2 | 1994003 | 615 | 3.1 | 13.5 | 53.9 | 2450.3 |
|  | Chr05A-C2 | 1769006 | 597 | 3.4 | 10 | 54.8 | 2440.0 |
|  | Chr06A | 2304633 | 678 | 2.9 | 19.2 | 57.6 | 2365.3 |
|  | Chr07A | 2331147 | 723 | 3.1 | 13.9 | 56.6 | 2390.2 |
|  | Chr08A | 1584971 | 511 | 3.2 | 14.8 | 56.8 | 2372.2 |
| Accessory | Chr09A | 862028 | 141 | 1.6 | 52.5 | 54.4 | 2240.1 |
|  | Chr10A | 827785 | 191 | 2.3 | 55.9 | 55.9 | 1991.4 |
|  | Chr11A | 426448 | 81 | 1.9 | 66.4 | 54.3 | 2001.7 |
|  | Chr04A-C1 | 500000 | 72 | 1.4 | 72 | 56.4 | 2165.4 |
|  | Chr05A-C1 | 590000 | 117 | 2.0 | 64.2 | 57.6 | 2102.0 |
| Core | Chr01B | 6594456 | 2130 | 3.2 | 12.9 | 56.6 | 2398.1 |
|  | Chr02B | 2497118 | 767 | 3.1 | 18.2 | 56.7 | 2367.3 |
|  | Chr03B | 2635414 | 834 | 3.2 | 14.4 | 56.6 | 2264.0 |
|  | Chr04B-C2 | 1905275 | 609 | 3.1 | 13.2 | 57 | 2416.6 |
|  | Chr05B-C1 | 3957202 | 1269 | 3.2 | 12 | 57 | 2066.2 |
|  | Chr06B | 2320739 | 689 | 3.0 | 19.3 | 56.5 | 2286.0 |
|  | Chr07B | 2210825 | 712 | 3.2 | 14.9 | 56.8 | 2309.6 |
|  | Chr08B | 1517388 | 494 | 3.3 | 11.7 | 57 | 2363.6 |
| Accessory | Chr09B | 852902 | 141 | 1.6 | 52.5 | 54.4 | 2240.1 |
|  | Chr10B | 810068 | 191 | 2.3 | 55.9 | 55.9 | 1991.4 |
|  | Chr11B | 427889 | 79 | 1.8 | 67.4 | 54.2 | 2145.5 |
|  | Chr12B | 1514773 | 483 | 3.2 | 16.4 | 56.9 | 2498.8 |
|  | Chr04B-C1 | 500000 | 82 | 1.6 | 65.1 | 54.3 | 2212.6 |
|  | Chr05B-C2 | 590000 | 103 | 1.7 | 67.1 | 54.7 | 2362.1 |

Table S6. Recombination information in each chromosome corresponding to Haplotype A genome.

| Chromosome | Recombination site number | Recombination event number | Recombination hotpot number (≧4 times) |
| --- | --- | --- | --- |
| Chr01A | 47 | 58 | 2 |
| Chr02A | 52 | 67 | 2 |
| Chr03A | 40 | 53 | 2 |
| Chr05A | 31 | 41 | 2 |
| Chr06A | 48 | 69 | 4 |
| Chr07A | 26 | 36 | 2 |
| Chr08A | 33 | 44 | 2 |
| Total | 277 | 368 | 16 |
