# Supplementary material for: Meiosis in an asymmetric dikaryotic genome of Tremella fuciformis Tr01 facilitates new chromosome formation
Source: Genome Biol. 2023 Dec 5;24:280. doi: 10.1186/s13059-023-03093-7 (PMC10696834; doi:10.1186/s13059-023-03093-7)
Supplement: Supplementary file 3 — Additional file 3. Review history. [file 13059_2023_3093_MOESM3_ESM.docx]

Review History

**First round of review**

**Reviewer 1**

**Were you able to assess all statistics in the manuscript, including the appropriateness of statistical tests used?**

No, I do not feel adequately qualified to assess the statistics.

**Comments to author:**

This is a very interesting study that presents the dikaryotic genome of an edible basidiomycete fungus. The group of researchers has presented extensive evidence about their findings, which include the presentation of the two-speed dikaryotic genome of the fungus, the structure of the genomes of several haploid spores produced after meiosis by the same strain and a suggested mechanism that is responsible for the aberrant genomic characteristics that these spores have. While the strain seems to be an unusual case, the study made me wonder how frequently such mechanisms are found in other basidiomycetes for which we know very little. I think the study is a nice contribution in understanding fungal genetics and genome architecture. At the same time because of the large number of different analyses the study requires careful reading. The figures are well presented and overall they help the reader to understand, but they are of low resolution in the pdf I used for the review, so please make sure the quality of the figures is good. From a methodology point of view I think the approach used here to sequence the two haploid genome of the dikaryon is very interesting.

Below I have comments that the authors can take into consideration.

I think parts of your introduction could be rearranged so that the text connects in a better way. At the end I got where you were heading, but for a good part of the introduction I was not sure how some of the statements connect e.g. the reference to Pleurotus.

Line 43. Natural seems to be an unnecessary word here.

Line 45. Citations are needed for the statement.

Lines 70-75. I was not sure how they follow the flow of your text.

Lines 83-85, you should report that this is a parasitic fungus. The word 'require' indicates some sort of symbiosis in general, but your reader should know that this is in fact a mycoparasite.

Line 89. What do you mean by connections? Citation?

Line 89. Why did you choose the word variety? Could you just use the word strain that we use in fungal biology more frequently?

Line 137. You mean meiosis.

Line 176 on instead of no.

Line 177. I think you meant to write supplementary figure 5.

Lines 237-239. Can you please explain what you think has taken place in this case?

Line 248. Shouldn't this be 2x?

Line 249. I am not sure I could connect the information you provide with your figure 4B.

Lines 339-340. Uncertain what you are trying to say here, please revise.

Line 340. Citation for the statement related to asthma?

Line 415-417. I think this is a statement for which there is not enough evidence in the study. One way to confirm this would be to try to understand the function (if known) of the genes that reside accessory compartments or chromosomes, but this has not been done here.

Lines 508-509. Why do you think the yeast form is unstable? And stable in what way? To be maintained in culture?

Lines 538. The word some is redundant. Why did you choose the mixed cultures since you could still isolate DNA from the yeast forms?

Fig S1 legend should be black not break.

Lines 567. Symbol for temperature needs to be fixed.

Line 653. It should be References.

Supplementary Figure 5. Please explain the values in y axis. (SNP density (1kb window))

Figure 2C. I am not sure the figure is exactly clear on the message you are trying to convey.

**Reviewer 2**

**Were you able to assess all statistics in the manuscript, including the appropriateness of statistical tests used?**

No, I do not feel adequately qualified to assess the statistics.

**Comments to author:**

In the manuscript by Deng, Guo, and Lin, et al., by applying PacBio long read HiFi sequencing, the authors first generated a complete genome assembly of a haploid basidiospore (DBZ04) isolated from a dikaryotic Tremella fuciformis strain Tr01. With this genome assembly as a reference, they next sequenced and assembled the two haploid genomes (A and B) in strain Tr01. By comparing the two nuclear genomes in the dikaryon Tr01, the authors identified and characterized regions that 1) had high/low similarities, 2) showed inter-chromosomal rearrangements, 3) were involved in homologous recombination. Additionally, they also identified 3 chromosomes and 2 chromosomal regions that had low gene densities and were enriched with repetitive sequences, which they termed as accessary chromosomes and chromosomal compartments. By Illumina sequencing of additional basidiospores from Tr01 and comparison to the parental strain, they found there was a biased inheritance of the haplotype A genome among the progeny, as well as the presence of two novel chromosomes (N1 and N2) in a subset of the basidiospores, potentially resulting from ectopic recombination during meiosis. The authors hypothesized that multiple chromosomes align and interact at their shared homologous regions during meiosis, and they proposed this as a model of how the observed chromosomal rearrangements and structural variations originated.

Major comments

1. The authors assert that they have assembled haploid genomes of the two nuclei in the dikaryon Tr01. However, the fact that they used DBZ04, a meiotic spore from Tr01, as the reference for phasing means the two haploid genomes that they generated may not represent the authentic genomes of the two nuclei in the dikaryon, because DBZ04 is a product of recombination. The authors in part acknowledged this in the text (line 110), but it is unclear why they still conclude that the DBZ04 genome "is nearly identical to one of the two nuclear genomes in Tr01". Additionally, consider phasing. While it is possible to delineate the two haplotypes of the same chromosome if there are sufficiently long sequencing reads bridging heterozygous regions, it is unclear if this can determine the association of alternative versions of different chromosomes. The DBZ04 genome is most likely a product of chromosome assortment, and thus, it will be a mixture of chromosomes from the two parental nuclei.

2. The authors state that they conducted PacBio HiFi sequencing of the dikaryon Tr01. Thus, if there is sufficient coverage, it would seem possible to phase the two nuclear genomes of the dikaryon based on this high-fidelity sequence, and thus to directly ascertain the genomes for the two independent nuclei harbored in the dikaryon. From the way the paper is written, it is unclear whether this approach was taken or if not, why not.

3. The finding of biased inheritance toward the haplotype A genome among basidiospores is interesting. However, given the issues regarding the genome assembly, it is not clear whether the data truly represent a biased inheritance of one nucleus over the other. They might still be consistent with basidiospores sharing higher than expected similarities, but the interpretation of the data could be very different. For example, what if the DBZ04 genome (and consequently haplotype A genome) represents some sequence combinations between the two nuclei that are favored among the meiotic products? One possible scenario could be due to divergence between the two nuclei and co-evolution, strong allele-dependent inheritance, or epistasis is established in one nucleus but not the other, which could potentially lead to a biased inheritance against the alleles that are involved in such interactions.

4. If the divergence between the two nuclei in the dikaryon Tr01 causes the low rate of sporulation observed in this strain, do mating between some of the basidiospores analyzed in this paper show enhanced sporulation, particularly the ones with higher sequence similarities? Also, it would be helpful if the authors could better explain how basidiospores were isolated from Tr01. It should be noted though that the presence of high sequence divergence and chromosome structural variation between the parental genomes do not necessarily lead to sporulation defects, such as during hybridization between species within, as well as closely related to, the pathogenic Cryptococcus species complex ( https://journals.asm.org/doi/full/10.1128/mbio.00764-19 and https://journals.plos.org/plosgenetics/article?id=10.1371/journal.pgen.1008871), where "novel" chromosomes resulting from ectopic recombination between diverging chromosomes also occurred.

5. The authors briefly described the identification of centromeres in the chromosomes assembled. A better description and characterization of these large repetitive-sequence-rich regions would be beneficial to the manuscript. Are any of these involved in the structural variation identified? Also, the authors stated that the two novel chromosomes both lack centromeres (line 281-282). How was this determined? Is it possible that they possess neo-centromeres? Additionally, regarding centromere mediated ectopic recombination, other than Candida and Malassezia, it was also first characterized in human fungal pathogens Cryptococcus neoformans and its closely related non-pathogenic species Cryptococcus amylolentus (https://journals.plos.org/plosgenetics/article?id=10.1371/journal.pgen.1004261 and https://journals.plos.org/plosbiology/article?id=10.1371/journal.pbio.2002527).

6. The process that the authors hypothesize, that genome rearrangements in a dikaryon lead to production of spores derived from only one of the two parental nuclei is surprisingly similar to a process that was recently discovered and reported in a publication in eLife. In that case, mating of two Cryptococcus isolates with differing karyotypes lead to the production of spores whose nuclear genome was derived from only one of the two parental genomes. The authors of the publication in eLife termed this pseudosexual reproduction. This should clearly be presented, discussed, cited in the context of the authors' studies here.

Uniparental nuclear inheritance following bisexual mating in fungi.

Yadav V, Sun S, Heitman J. Elife. 2021 Aug 2;10:e66234. doi: 10.7554/eLife.66234. PMID: 34338631 Free PMC article.

Minor comments

1. The manuscript would benefit from polishing. Below please find some minor edits on the text, but they are not comprehensive.

2. Line 36. Maybe "infertility" should be "inviability"?

3. Line 62-64. The "MAT", "P/R", and "HD" should be italic. Same comment for the rest of the manuscript.

4. Line 68. "plasmogamy" should be "karyogamy".

5. Line 86. I appreciate what the authors are trying to convey here, but I don't think strictly speaking Cryptococcus neoformans is considered a dimorphic fungus.

6. Line 126. It says here "eight large regions" but in Figure S5 there are 9 highlighted regions.

7. Line 176. Should the "no" be "on" here? It is currently confusing.

8. Line 188. Add "in" following "type loci".

9. Line 192-193. "SIX1" and "SIX2" should be "SXI1" and "SXI2", respectively, and italic. Same for Figure S8, the gene names should all be italic.

10. Line 198-202. For those P values, what are the statistic tests?

11. Line 237-238. Why "after meiosis"? It seems an at least equally plausible explanation could be a compromised chromosome assortment during meiosis.

12. Line 251. "1 515,015" should be "1,515,015".

13. Line 273. Should "becomed" be "become" or "became"?

14. Line 277. If the novel chromosome N1 is only 0.23 Mb, can you see it on a CHEF gel?

15. Line 291. Why only "meiosis" here? Wouldn't similar events occur during mitosis as well?

**Reviewer 3**

**Were you able to assess all statistics in the manuscript, including the appropriateness of statistical tests used?**

No, I do not feel adequately qualified to assess the statistics.

**Comments to author:**

The paper by Deng and colleagues report the genome acquisition and phasing of a dikaryotic basidiomycetes. The authors provide good quality genome data on this organisms.

I have no particular concern with regards to their results or interpretation of the data. I think the study is of sufficient quality to be published pending revisions and additional work to back up some of the claims

Furthermore, I do have significant concerns with regards to the "perceived impact" of this study, in particular on whether this work is really NOVEL and/or provides new METHODS to phase complex data. I do not think this is the case given available literature.

My main concerns what this study brings in terms of technical novelty that would allow, for example, to fully assembly a complex genome without the use of HiFi and HiC. This has not been proven here because these techniques were not uses independently to assemble the data, and there is not evidence that those genomes could not have been generated using only Hi-C and HiFi without a references.

Specific comments:

Abstract:

"

"Accurate phasing of these two nuclear genomes and their interactions have long been of interest but remain mostly unknown"

This is arguably false. Significant work that produced fully phased genome data and analyses from dikaryotic genomes that are even larger than the one analyses here have been published, including in this journal.

See H Duan et al. 2022, EC Henningse et al 2022, J Sperschneider et al 2021 B Schwessinger et al. 2021 to name a few...

L49: "For example, aneuploidy and isochromosome formation in pathogenic Candida albicans".

This example has nothing to do with rearrangments...

L92 (and elsewhere): In the present study, we used a "nucleus-by-nucleus strategy"

Please define. This is very vague terminology that is used multiple times in this paper but has never been defined. It sounds cool, but ultimately what the authors did is using monokaryon reference and HiFi and HiC data. How is this a nucleus-by-nucleus approach?

L94: "in which recombining regions were replaced by allelic sequences to assemble the complete genome of one nucleus."

So you are creating new (potentially artefactual) assemblies ? This is very confusing for the reader. Please rephrase for clarity.

L123: "We adopted a nucleus-by-nucleus strateg ".

This means nothing, really. Also, why using a convoluted and badly define approach instead of simply using HiFi to build scaffolds and HiC to obtain full chromosomes. HiFiasm has a MODE that uses Hi-C reads. This should be used here.

L145: "Hi-C analyses showed that contacts among chromosomes within the same nucleus were stronger than those between two nuclei":

need to show data/figure. In fact, authors should use the Nuclear Phaser pipeline from Daur et el 2022 Genome Biology to test this.

L160-161:

This is sentence is confusing. How does telomere to telomere interactions suggests Hap1/2 are incorrect?

Please rephrase.

Also on this, any evidence for the presence of A/B compartments in this genome, as seen in Epichloe and more recently in AMF(Winter 2018., Yildirir 2022) ? Looking at your HiC maps, it looks like you have compartimentization ongoing.

L218-220: The authors should determine the rate of rearrangements on these regions compared to the core genome, and see a significant increase is variability

L294-296: These two paragraphs contain conclusive statements about how events may have occured. However, these are still speculations based on genomics and until empirical evidence is provided the authors should be careful in their interpretations and use "conditional" sentences explain these events.

I suggest the authors to use this part of the paper to build a model that explain what may have caused this event. A detailed figure explaining the model should be provided

L339-340: "With this strain, less production lossed due to basidiosporejection were reduced!"

Please rephrase

L368: The results section contains a lot of discussion, so either mesh these into one results and discussions section or clearly separate both?

In any case, please make this much more concise.

L376: "However, phasing of haplotypes within a dikaryotic genome still remains challenging."

yes, it is challenging but it is feasible with HiFi + HiC and the current paper doesn't demonstrate how their approach is better at achieving this.

L389: "false positive fusions of allelic contigs that can result with current scaffolding methods[26]"

The authors actually never demonstrated that their phases are correct, and do not contain phase swaps for example. See Duan et al. 2022

L432-438: no need to detail all the resuts again, Provide a good figure and legend that clearly explains this instead

**Authors Response**

**Point-by-point responses to the reviewers’ comments:**

**Reviewer#1**

The figures are well presented and overall they help the reader to understand, but they are of low resolution in the pdf I used for the review, so please make sure the quality of the figures is good.

***Response:*** *Thanks very much for reviewing our manuscript. All figures have been made with high resolution. We will pay attention to the resolution of converted PDF files before uploading them.*

**Q2：**I think parts of your introduction could be rearranged so that the text connects in a better way. At the end I got where you were heading, but for a good part of the introduction I was not sure how some of the statements connect e.g. the reference to *Pleurotus*.

***Response:*** *We revised and reorganized the introduction section, and removed the contents related to Pleurotus. The statement “For many fungi belonging to the basidiomycetes, sexual compatibility is determined…” was revised into “For many fungi belonging to the basidiomycetes, such as T. fuciformis, sexual compatibility is determined…(Lines 86-87)”.*

**Q3:** Line 43. Natural seems to be an unnecessary word here.

***Response:*** *Revised.*

**Q4:** Line 45. Citations are needed for the statement.

***Response:*** *Added.*

**Q5:** Lines 70-75. I was not sure how they follow the flow of your text.

***Response:*** *We removed this part.*

**Q6:** Lines 83-85, you should report that this is a parasitic fungus. The word 'require' indicates some sort of symbiosis in general, but your reader should know that this is in fact a mycoparasite.

***Response:*** *Although many people think the relationship between T. fuciformis and Annulohypoxylon stygium is parasitism, based on our research in many years, T. fuciformis does not pose a threat to A. stygium. We have been searching for evidences that the existence of T. fuciformis could be beneficial to A. stygium. For example, higher drought tolerance properties of T. fuciformis may allow their complexes to live longer under drought conditions (unpublished). That is the reason we have been vague on this subject since our data have not been published yet.*

**Q7：**Line 89. What do you mean by connections? Citation?

***Response:*** *Mycelia of T. fuciformis is able to physically contact mycelia of A. stygium, and absorbs nutrition from A. stygium. During the cultivation of T. fuciformis, the spawn is composed of T. fuciformis and A. stygium mycelia, and does not include T. fuciformis yeasts. There is no related literature available.*

**Q8:** Line 89. Why did you choose the word variety? Could you just use the word strain that we use in fungal biology more frequently?

***Response:*** *You are right and the word strain is more appropriate. The sentence was revised as “Tr01 is a common strain of T. fuciformis wildly cultivated in China, forming white fruiting bodies.(Lines 82-83)”*

**Q9:** Line 137. You mean meiosis.

***Response:*** *Revised.*

**Q10:** Line 176 on instead of no.
***Response:*** *Revised.*

**Q11:** Line 177. I think you meant to write supplementary figure 5.

***Response:*** *Revised.*

**Q12:**Lines 237-239. Can you please explain what you think has taken place in this case?

***Response:*** *Genomes of DBZ03, DBZ23, DBZ25, DBZ29 carried non integer multiple copies of Chr11, indicating that cells sequenced contained two or more karyotypes and each of which carries different copies of Chr11. All the cells germinated from a single basidiospore. So gain or loss of whole Chr11 occurred after meiosis. For better understanding of readers, we revised “The best explanation for this phenomenon is that these gains or losses occurred during division of some yeast-like cells after meiosis.” as “Germinated from a single basidiospore, sequenced genomes should be two or more karyotypes, each of which carried different copies of Chr11, indicating that gain or loss of whole Chr11 occurred in some cells after meiosis. (Lines 297-299)”*

**Q13:** Line 248. Shouldn't this be 2x?

***Response: Yes,*** *revised.*

**Q14:** Line 249. I am not sure I could connect the information you provide with your figure 4B.

***Response:*** *For better understanding, we added a sentence. “In DBZ02, 2.0× more sequence read depth was detected in regions of Chr05B:1–675,684 and Chr12B:1–1,515,015 than in DBZ43 (Figure 4B).” was revised into “In DBZ02, PacBio long reads supported the connection of upstream and downstream sequences at each of sequencing depth inflection points A and B (Figure 4B). Furthermore, 2.0× more sequence read depth was detected in regions of Chr05B:1–675,684 and Chr12B:1–1,515,015 than in DBZ43. (Lines 313-314)”.*

**Q15:** Lines 339-340. Uncertain what you are trying to say here, please revise.

***Response:*** *It was an interpretation sentence in Results section. We moved it to the discussion section.* ***“…****because massive production of spores can cause many serious problems, including respiratory allergic reactions to workers[31, 32] and mushroom production losses. (Lines 621-622)****”.***

**Q16:** Line 340. Citation for the statement related to asthma?

***Response:*** *Added as shown in response above.*

**Q17:** Line 415-417. I think this is a statement for which there is not enough evidence in the study. One way to confirm this would be to try to understand the function (if known) of the genes that reside accessory compartments or chromosomes, but this has not been done here.

***Response:*** *We rephrased this sentence. “Thus, we propose that the accessory chromosomes/compartments in T. fuciformis might harbor genes associated with its interaction with A. stygium.” was revised into “It provided evidence that the accessory chromosomes/compartments in T. fuciformis might be associated with its interaction with A. stygium. (Lines 506-508)”.*

**Q18:** Lines 508-509. Why do you think the yeast form is unstable? And stable in what way? To be maintained in culture?
Lines 538. The word some is redundant. Why did you choose the mixed cultures since you could still isolate DNA from the yeast forms?

***Response:*** *The word some is deleted. T. fuciformis Tr01 has been cultivated stably in China for more than 30 years. Its spawn is composed of mycelia of A. stygium and T. fuciformis. So the mycelial type of Tr01 is stable.*

*We isolated 30 single yeast strains of dikaryon Tr01, did genome sequencing for each isolates, and mapped sequencing reads on Haplotype A genome respectively. Results showed genome sequence* *of each single yeast isolates (Y14-Y106) were different from each other (see Figure below), indicating that yeast type of Tr01 is unstable. Mycelial type of Tr01 are stable (M1-M5). So we used mycelial cells from mixed cultures for DNA isolation.*

*Figure 1. Genome-wide distribution of GVs in five mycelial strains (M1-M5) and 30 yeast isolates (Y14-Y106). C1-C11 represent Chr01A to Chr11A of Tr01 genome. One bar represents 100-kb region. Blue, CNV region; yellow, LOH region; red, hemizygosity region; grey, unchanged region. Within one bar, length ratio of one color to whole bar represents percentage of corresponding GVs.*

**Q19:** Fig S1 legend should be black not break.

***Response:*** *Revised accordingly.*

**Q20:** Lines 567. Symbol for temperature needs to be fixed.
***Response:*** *Done.*

**Q21:** Line 653. It should be References.

***Response:*** *Revised.*

**Q22:** Supplementary Figure 5. Please explain the values in y axis. (SNP density (1kb window))
***Response:*** *Revised.*

**Q23:** Figure 2C. I am not sure the figure is exactly clear on the message you are trying to convey.
***Response:***  *We rephrased the sentence Lines 157-159 (before revised version). “Heat maps derived from Hi-C data also supported the linkage of upstream and downstream sequences of each breakpoint (Figure 2C and Supplementary Figure 6).” was revised as “*Strong Hi-C signals between *upstream and downstream sequences of each breakpoint (B and J shown in Figure 2C, others shown in Supplementary Figure 7) also supported their neighbor relationship.(Lines 203-205)”.*

**Reviewer#2**

**Q1:** The authors assert that they have assembled haploid genomes of the two nuclei in the dikaryon Tr01. **Q1-1)** However, the fact that they used DBZ04, a meiotic spore from Tr01, as the reference for phasing means the two haploid genomes that they generated may not represent the authentic genomes of the two nuclei in the dikaryon, because DBZ04 is a product of recombination. **Q1-2)** The authors in part acknowledged this in the text (line 110), but it is unclear why they still conclude that the DBZ04 genome "is nearly identical to one of the two nuclear genomes in Tr01". **Q1-3)** Additionally, consider phasing. While it is possible to delineate the two haplotypes of the same chromosome if there are sufficiently long sequencing reads bridging heterozygous regions, it is unclear if this can determine the association of alternative versions of different chromosomes. The DBZ04 genome is most likely a product of chromosome assortment, and thus, it will be a mixture of chromosomes from the two parental nuclei.

***Response:***

***1-1)*** *DBZ04 is a meiotic spore from Tr01. Its genome consists of sequences from non-recombinant regions of one nuclear genome and recombinant regions of the other. During assembling each nuclear genome in T. fuciformis Tr01,we detected all possible recombination breakpoints occurred in DBZ04 using homologous recombination (HR) contigs (Lines 174-175, Supplementary Figure 4 A and D), and replaced all recombinant regions by allelic sequences to form Haplotype A and B nuclear genomes, respectively (Lines 181-183). Therefore, Tr01-Haplotype A and B genomes represent the authentic genomes of the two nuclei in the dikaryon Tr01. Compared with Tr01-Haplotype A genome, we detected three recombinant regions in DBZ04 genome, including one inside Chr01, the other two near the ends of Chr05 and Chr07 (Lines177-179).*

***1-2）****DBZ04 genome consists of sequences from non-recombinant regions of one nuclear genome and recombinant regions of the other. The phrase in line110-111 “the DBZ04 genome is nearly* *identical to one of the two nuclear genomes in Tr01 except for some regions of recombination” may be inaccurate. We revised “A haploid genome derived from T. fuciformis* *Tr01, DBZ04, was required as a reference for assembling both of its complete dikaryotic haploid genomes. Because DBZ04 is a monokaryotic isolate that originates from a germination of a single Tr01 basidiospore, the DBZ04 genome is nearly identical to one of the two nuclear genomes in Tr01 except for some regions of recombination.” as “A haploid genome derived from T. fuciformis* *Tr01, DBZ04, was assembled first to be used as a reference genome to separate the two dikaryotic haploid genomes. DBZ04 was a monokaryotic isolate originated from the germination of a single Tr01 basidiospore.”.*

***1-3)*** *One unusual characteristics of T. fuciformis Tr01 is its asymmetric dikaryotic genome, in which the sequences flanking six breakpoints in the Tr01-Haplotype B genome showed non-collinear arrangements with the Tr01-Haplotype A genome, revealing five structural variants between these two nuclear genomes. Four structural variants associated with different chromosomes. We assessed these associations cautiously as follow: 1) mapping all contigs on the reference genome to search possible contigs showing inter-chromosomal arrangements (CR contigs, Supplementary Figure 4E). 2) PacBio HiFi reads mapping on association breakpoints of CR region (Figure 2A, an detail example shown in Figure 2B) to verify assembled correction of the CR regions. 3) Analyses of repetitive sequences of CR regions (Figure 2B) were performed to verify that the CR contigs result from association of different chromosomes, rather than insertion of transposons. 4) Hi-C analyses were performed to confirm these associations of different chromosomes (Figure 2C and Supplementary Figure 6). Mutual authentication of PacBio HiFi data and Hi-C analyses ensure accurate genome assembly, including structural variant regions.*

**Q2:** The authors state that they conducted PacBio HiFi sequencing of the dikaryon Tr01. Thus, if there is sufficient coverage, it would seem possible to phase the two nuclear genomes of the dikaryon based on this high-fidelity sequence, and thus to directly ascertain the genomes for the two independent nuclei harbored in the dikaryon. From the way the paper is written, it is unclear whether this approach was taken or if not, why not.

***Response:*** *We did assemble two nuclear genomes of the dikaryon Tr01 using PacBio HiFi reads. The assemblies are high quality, but not two completed nuclear genomes. The reasons are: 1) Tr01 dikaryotic genome contains many regions of low heterozygosity (Supplementary Figure 5), some of which have less than 0.1 % SNP density and are larger than the length of the HiFi reads. Examples are Chr02A:1800kb-2000kb, Chr05A:1bp-400kb, and Chr07A:1000kb-1200kb (Supplementary Figure 5). 2) It is hard to phase chromosomes into different nuclear genomes using PacBio HiFi data.*

*Here, we generated two nuclear genomes of the dikaryon Tr01 using monokaryon reference genome and HiFi and Hi-C data. A monokaryon reference genome made it possible to solve* *phasing problems at low heterozygosity regions. Duo to physical separation of haplotypes in dikaryons, unlike in diploids, Hi-C chromatin contacts between haplotypes are false-positive signals. Hi‑C data are able to phase chromosomes into different haplotype genomes.*

**Q3：**The finding of biased inheritance toward the haplotype A genome among basidiospores is interesting. However, given the issues regarding the genome assembly, it is not clear whether the data truly represent a biased inheritance of one nucleus over the other. They might still be consistent with basidiospores sharing higher than expected similarities, but the interpretation of the data could be very different. For example, what if the DBZ04 genome (and consequently haplotype A genome) represents some sequence combinations between the two nuclei that are favored among the meiotic products? One possible scenario could be due to divergence between the two nuclei and co-evolution, strong allele-dependent inheritance, or epistasis is established in one nucleus but not the other, which could potentially lead to a biased inheritance against the alleles that are involved in such interactions.

***Response:*** *Following your comments, we reassessed our interpretation. Among 33 monospore isolates studied, 17 had Tr01-Haplotype A architecture with 11 chromosomes, 11 isolates had 12 chromosomes, and three isolates had 13 chromosomes. Eight chromosomes shared high sequence collinearity between Tr01-Haplotypes A and B, including Chromosomes 3, 4, and 6 to 11. Three chromosomes contained rearrangements and sequence variation, including chromosomes 1, 2, and 5 between the two haplotypes (Figure 1). Chromosomes 12B, N1, and N2 were extra chromosomes. Based on the analysis of the three variable chromosomes, 31 of the 33 monospore isolates contain Tr01-Haplotype A architecture, showing a biased inheritance. Moreover, there are other two peculiar features of basidiospores of T. fuciformis Tr01. The mature fruiting bodies produced numerous four-celled phragmobasidia with cruciate septa, from which few basidiospores generated. 14 monospore isolates (42.4 %) were aneuploids with one or two extra chromosomes. The scenario that epistasis is established in one nucleus won’t explain the two phenomena.*

*In the study, we proposed a scenario based on meiosis analyses of the asymmetric dikaryotic genome. The scenario is able to provide proper explanation for all the three phenomena related to basidiospores of Tr01. Here are the details (Lines 572-601):*

*“During prophase I of meiosis, homologous regions among T. fuciformis Tr01 Chr01A, Chr01B, Chr02A, Chr02B, Chr05A, Chr05B, and Chr12B paired up to form a large homologous complex, leading to some inter- and intra-chromosomal, physical contacts (Supplementary Figure 11A left). These contacts facilitated abnormal rearrangements within or between these chromosomes, which might result in uneven distribution of genetic materials into daughter cells and fragmented chromosomes (Supplementary Figure 11A middle). Uneven distribution of genetic materials led to one or both daughter cells lacking some core genes essential for their survival. Fragmented chromosomes were unstably inherited and are gradually lost, leading to the inviability of some daughter cells (Supplementary Figure 11A right).*

*During metaphase I, each chromosome was able to migrate into either daughter cells by spindle fibers (Supplementary Figure 11B left). However, disruption of combination of Chr01A, Chr02A and Chr05A (Tr01-Haplotype A architecture) resulted in daughter cells lacking essential genes for survival. For example, exchange of just Chr01 occurring during metaphase I process can result in two different types of daughter cells: type A lacking a fragment of Chr01A:1-211832 (including 148 BUSCOs) and type B containing a pair of the homologous sequences (Supplementary Figure 11B right).* [*Moreover*](javascript:;)*, type B daughter cells tended to lose other core genes duo to free movement of Chr12B. As a result, few basidiospores are able to survive, most of which are characterized by Tr01-Haplotype A architecture.*

*Tr01-Haplotype B genome included a core chromosome Chr12B that lacked a centromere. Without the ability to attach to kinetochore microtubules, Chr12B was more likely to migrate to daughter cells with Tr01-Haplotype A architecture following its paired Chr05A during meiosis (Supplementary Figure 11C left). Chr12B contained 103 BUSCOs, at least 80 of which were required for monospore isolate survival. As a result, many monospore isolates with Tr01-Haplotype A architecture contained an extra Chr12B (Supplementary Figure 11C right, 33%) and few monospore isolates (6%) exhibited Tr01-Haplotype B architecture. Accordingly, isolates with Tr01-Haplotype A architecture contained more sequences deriving from Tr01-Haplotype A genome. Similarly, some monospore isolates contain an extra ChrN1 or ChrN2”*

*Thus, the scenario we provided may be more suitable for T. fuciformis Tr01.*

**Q4:** If the divergence between the two nuclei in the dikaryon Tr01 causes the low rate of sporulation observed in this strain, **Q4-1)** do mating between some of the basidiospores analyzed in this paper show enhanced sporulation, particularly the ones with higher sequence similarities? **Q4-2)** Also, it would be helpful if the authors could better explain how basidiospores were isolated from Tr01. **Q4-3)**It should be noted though that the presence of high sequence divergence and chromosome structural variation between the parental genomes do not necessarily lead to sporulation defects, such as during hybridization between species within, as well as closely related to, the pathogenic Cryptococcus species complex ( https://journals.asm.org/doi/full/10.1128/mbio.00764-19 and https://journals.plos.org/plosgenetics/article?id=10.1371/journal.pgen.1008871), where "novel" chromosomes resulting from ectopic recombination between diverging chromosomes also occurred.

***Response: 4-1)*** *It is can be a good way to enhance sporulation by mating of basidiospores with high sequence similarities. Mating between two basidiospore isolates can form a yeast-type dikaryotic strain. Mycelial type of dikaryon Tr01 is required to produce its fruiting bodies. We tried many times to transform yeast type of Tr01 strain into mycelial type, but failed. We isolated 30 single yeast strains of dikaryon Tr01, performed genome sequencing for each isolate, and mapped sequencing reads on Tr01-Haplotype A genome respectively. Results showed genome sequence of each single yeast isolates (Y14-Y106) were different from each other, indicating that yeast type of Tr01 is unstable. Mycelial type of Tr01 are stable (M1-M5). Unstable genome may be a reason to* *hinder its transform from yeast to mycelium. At present, it is unsuitable to do mating work for the strain Tr01.*

*Figure 1. Genome-wide distribution of GVs in five mycelial strains (M1-M5) and 30 yeast isolates (Y14-Y106). C1-C11 represent Chr01A to Chr11A of Tr01 genome. One bar represents 100-kb region. Blue, CNV region; yellow, LOH region; red, hemizygosity region; grey, unchanged region. Within one bar, length ratio of one color to whole bar represents percentage of corresponding GVs.*

***4-2)*** *We added the process of monospore insolation of Tr01 (Strains section in Materials and Methods), as following: “For monospore isolation, a piece of matured fruiting body was suspended in autoclaved conical flask with stopper two days. Basidiospores were collected by bottom washing of autoclaved water, and observed under* [*optical microscope*](javascript:;)*. After* [*serial dilution*](javascript:;)*, plate cultivation and streak plate, single colony in each plate was isolated as a monospore isolate. (Lines 669-673)”*

***4-3)*** *As shown in the article (https://journals.asm.org/doi/full/10.1128/mbio.00764-19), “while many crosses had low rates of spore production, we specifically collected progeny from 2 and 3 of the DSM …” (page 12, paragraph 2, line 4) and “The average germination rate per basidium for each cross ranged from 1% to 22% (Table 2)” (page 6, paragraph 2, line 8-9). In the article (https://journals.plos.org/plosgenetics/article?id=10.1371/journal.pgen.1008871), “Dissected basidiospores from wild-type H99α (C. neoforman) x JEC20 (C. deneoformans) acrosses germinated at a frequency of 5.8%, which is similar to the previously published frequency of 5%. “(page 6, paragraph 3, line 6-7). Although hybridization between closely related species mentioned* *above articles do not lead to sporulation defects, but have low rates of spore production and germination, showing same tendency with Tr01.*

**Q5:** The authors briefly described the identification of centromeres in the chromosomes assembled. **Q5-1）**A better description and characterization of these large repetitive-sequence-rich regions would be beneficial to the manuscript. Are any of these involved in the structural variation identified? **Q5-2）**Also, the authors stated that the two novel chromosomes both lack centromeres (line 281-282). How was this determined? Is it possible that they possess neo-centromeres? **Q5-3）**Additionally, regarding centromere mediated ectopic recombination, other than Candida and Malassezia, it was also first characterized in human fungal pathogens Cryptococcus neoformans and its closely related non-pathogenic species Cryptococcus amylolentus (https://journals.plos.org/plosgenetics/article?id=10.1371/journal.pgen.1004261 and https://journals.plos.org/plosbiology/article?id=10.1371/journal.pbio.2002527).

***Response: 5-1)*** *we revised the Supplementary Table 1: add information of Tcn1 and its remnants, add proportion for three types of common transposons.*

*“One centromere for each chromosome was detected by Hi-C mapping (Supplementary Figure 3). All of these centromeres (40-Kb resolution) contained retroelements and their remnants including multiple copies of Tcn6 or rnd-4_family-1813 transposons (except for Chr10, Supplementary Table 1).” was revised into “One potential centromere region (40 kb per region) for each chromosome was predicted by Hi-C mapping (Supplementary Figure 3). Each region was unique but all were rich in repeat elements, occupying 69.5 % to 100 % of whole regions (Supplementary Table 1). All regions except for that of Chr10 contained at least two copies of Tcn6 transposon, except for those of Chr01 and Chr10 contained at least one copy of Tcn1 transposon. Both Tcn1 and Tcn6 transposons are commonly found in centromeres of Cryptococcus species complex[**18**], which belong to Tremellaceae, in the same order with T. fucifomis. Rnd-4 family-1813, belonging to the Ty1-copia family of retroelements, was also found in most centromere regions of DBZ04 genome.(Lines 152-160)”.*

*Locations of each centromere (red bars in outer ring) and each breakpoint of structural variation (black arrowheads) can be found in Figure 1. All breakpoints are far from centromeres, indicating that none of the centromeres are involved in the structural variations. In legend of Figure 1, we add a sentence “Red bars in outer ring represent centromere locations of each chromosome. (Second sentence)”.*

***5-2)*** *Chr12B was stated to be a centromere free chromosome based on following evidences: 1) No centromeric Hi-C signal were detected in the Chr12B; 2) about one third of monospores contained an extra Chr12B, indicating Chr12B can be randomly sorted into either daughter cell during meiosis without traction of spindle fibers. Chr12B has existed in genome of dikaryon Tr01 for a long time, indicating new centromere is not easy to form in the strain.*

*ChrN1 is a newly formed chromosome connecting Chr01B:1–58690 and reverse complementary of Chr12B:1–172535. Both regions didn’t contain centromeric DNA sequences in haplotype B genome. Thus, we inferred ChrN1 as a centromere free chromosome. It is the same for ChrN2.*

*Lines 281-282 (old version), “Both new chromosomes were capped by telomeres at both ends, but do not have centromeres” was revised into “Due to each of component segments containing one telomere and no centromere, both new chromosomes were capped by telomeres at both ends, but did not have centromeres.(Lines 346-348)”.*

***A5-3)*** *We added the information related to Cryptococcus amylolentus in the introduction section. “Such centromere-mediated chromosome rearrangements have also been detected in, Candida and Malassezia.” was revised into “Centromere-mediated chromosome rearrangements have also been detected in C. amylolentus, Candida and Malassezia. (Lines 64-66)”.*

**Q6)** The process that the authors hypothesize, that genome rearrangements in a dikaryon lead to production of spores derived from only one of the two parental nuclei is surprisingly similar to a process that was recently discovered and reported in a publication in eLife. In that case, mating of two Cryptococcus isolates with differing karyotypes lead to the production of spores whose nuclear genome was derived from only one of the two parental genomes. The authors of the publication in eLife termed this pseudosexual reproduction. This should clearly be presented, discussed, cited in the context of the authors' studies here.

***Response:*** *C. neoformans VYD135α and KN99a cross lead to production of spores derived from only one of the two parental nuclei. The uniparental progenies lack nuclear recombination between two parents. However, basidiospores of Tr01 produce recombinant progenies, and contain genetic materials from both parental nuclei. Formation mechanism of these two basidiospores are different.*

*We added the publication in eLife (https://elifesciences.org/articles/66234) in the discussion section. “Generally, numerous basidiospores can be ejected from fruiting bodies, and have no bias towards either parental nucleus in genetic material. However, there are exceptions. In the case of C. neoformans, hybridization of two isolates with differing karyotypes (VYD135α and KN99a) lead to production of spores whose nuclear genomes were derived from only one of the two parental genomes[30]. The pseudosexual reproduction referred was supposed to stem from nuclear loss via hyphal branches. (Lines 559-564)”.*

**Minor comments**
**Q7:**  The manuscript would benefit from polishing. Below please find some minor edits on the text, but they are not comprehensive.

***Response:*** *We went over the manuscript carefully and corrected minor errors.*

**Q8:** Line 36. Maybe "infertility" should be "inviability"?

***Response:*** *Revised.*

**Q9:** Line 62-64. The "MAT", "P/R", and "HD" should be italic. Same comment for the rest of the manuscript.

***Response:*** *Revised.*

**Q10:** Line 68. "plasmogamy" should be "karyogamy".

***Response:*** *Here, our meaning here is “the cytoplasm of two parental mycelia fuse together without the fusion of nuclei.” So we think here should be "plasmogamy" but not "karyogamy".*

**Q11:** Line 86. I appreciate what the authors are trying to convey here, but I don't think strictly speaking Cryptococcus neoformans is considered a dimorphic fungus.

***Response:*** *We deleted the sentence “Similar to other species in the Tremellaceae, such as the human pathogen C. neoformans,”*

**Q12:** Line 126. It says here "eight large regions" but in Figure S5 there are 9 highlighted regions.

***Response:*** *Revised. It should be “nine large regions”.*

**Q13:** Line 176. Should the "no" be "on" here? It is currently confusing.

***Response:*** *Revised.*

**Q14:** Line 188. Add "in" following "type loci".

***Response:*** *Revised.*

**Q15:** Line 192-193. "SIX1" and "SIX2" should be "SXI1" and "SXI2", respectively, and italic. Same for Figure S8, the gene names should all be italic.

***Response:*** *Revised.*

**Q16:** Line 198-202. For those P values, what are the statistic tests?

***Response:*** *One-way ANOVA, added.*

**Q17:** Line 237-238. Why "after meiosis"? It seems an at least equally plausible explanation could be a compromised chromosome assortment during meiosis.

***Response:*** *Genomes of DBZ03, DBZ23, DBZ25, DBZ29 carried non integer multiple copies of Chr11, indicating that the sequenced genomes contained two or more types, each of which carries different copies of Chr11. All the cells germinated from a single basidiospore. So gain or loss of whole Chr11 occurred after meiosis. To make it clearer, we revised “The best explanation for this phenomenon is that these gains or losses occurred during division of some yeast-like cells after meiosis.” as “Germinated from a single basidiospore, sequenced genomes should be two or more karyotypes, each of which carried different copies of Chr11, indicating that gain or loss of whole Chr11 occurred in some cells after meiosis. (Lines 297-299)”.*

**Q18:** Line 251. "1 515,015" should be "1,515,015".

***Response:*** *Revised.*

**Q19:** Line 273. Should "becomed" be "become" or "became"?

***Response:*** *Revised.*

**Q20:** Line 277. If the novel chromosome N1 is only 0.23 Mb, can you see it on a CHEF gel?

***Response:*** *It is a good way to determine a small chromosome using method combining CHEF technology and southern blot. The first step for CHEF gel making is to obtain enough protoplasts of monospore isolates. Duo to Tr01 cells containing capsule, we have tried several times, but failed to obtain their protoplasts even by the method used in Cryptococcus neoformans. So we didn’t get a CHEF gel image of this N1 chromosome.*

**Q21:** Line 291. Why only "meiosis" here? Wouldn't similar events occur during mitosis as well?
***Response:*** *You are correct and similar events of random assortment of these three chromosomes without centromere occur during mitosis. We did add mitosis to the last sentence in conclusion* *below the Discussion as below, thanks to your comments:*

*“Irregular migration during meiosis and mitosis of centromere-free chromosomes such as Chr12B, ChrN1, and ChrN2 resulted in aneuploidy of some daughter cells and inviability of most other daughter cells. (Lines 661-663)”.*

*However, in line 291 of the previous version, we were discussing the formation of three new chromosomes without centromere combining DNA fragments from Tr01-Haplotypes A and B, which occurs only in meiosis. We revised and moved this paragraph to the Discussion section as copied below:*

*“Tr01-Haplotype B genome includes an accessary chromosome Chr12B that lacks a centromere. Without the ability to attach to kinetochore microtubules, Chr12B is more likely to migrate to daughter cells with Haplotype A architecture following its pairing with Chr05A during meiosis (Supplementary Figure 11C left). Chr12B contains 103 BUSCOs, at least 80 of which are required for monospore isolate survival. As a result, many monospore isolates with Tr01-Haplotype A architecture contain an extra Chr12B (Supplementary Figure 11C right, 33%) and few monospore isolates (6%) exhibited Tr01-Haplotype B architecture. Accordingly, isolates with Haplotype A architecture contain more sequences deriving from Tr01-Haplotype A genome. Similarly, some monospore isolates contain an extra ChrN1 or ChrN2. (Lines 592-601)”*

**Reviewer#3**

**Q1:** The paper by Deng and colleagues report the genome acquisition and phasing of a dikaryotic basidiomycetes. The authors provide good quality genome data on this organisms.

I have no particular concern with regards to their results or interpretation of the data. I think the study is of sufficient quality to be published pending revisions and additional work to back up some of the claims

Furthermore, I do have significant concerns with regards to the "perceived impact" of this study, in particular on whether this work is really NOVEL and/or provides new METHODS to phase complex data. I do not think this is the case given available literature.

My main concerns what this study brings in terms of technical novelty that would allow, for example, to fully assembly a complex genome without the use of HiFi and HiC. This has not been proven here because these techniques were not uses independently to assemble the data, and there is not evidence that those genomes could not have been generated using only Hi-C and HiFi without a references.

***Response:***

*Dikaryotic genome of T. fuciformis Tr01 is not big, but complex. It is a genome with various heterozygosity, including some low heterozygosity regions (~32.4 % of the genome < 0.7 % heterozygosity), some middle heterozygosity regions (e.g. Chr05:600Kb-1600Kb), and some high heterozygosity regions. NuclearPhaser (H Duan et al. 2022) is the first Hi-C based phasing pipeline to phase dikaryon, and can be used to phase genomes of ~0.7 % heterozygosity with ~30-40× haploid genome coverage. Moreover, for the cases of low heterozygosity regions interspersed by middle or high heterozygosity ones (e.g. Chr02:1800kb-2400kb), phasing using Hi-C and HiFi data may not work well. There are several inter- and intra- chromosome rearrangements between the two nuclear genomes. Without a reference genome, it is uncertain if there are chromosome rearrangements. We did assemble the dikaryontic genome of T. fuciformis Tr01 using only Hi-C and HiFi without a references. The result was not good enough.*

*In this manuscript, we generated a high-quality genome sequence of its own single basidiospore isolate of Tr01. Using the genome as reference, we assembled one nuclear genome showing identical genomic architecture and high sequence similarity to the reference at first, and generated the other genome using the remainder contigs, referred as "nucleus-by-nucleus strategy".*

*For many basidiomycetes, single basidiospore isolate is readily accessible. It is not hard to generate monokaryon genome reference of high quality. Monokaryon reference can promote the accuracy and reliability of the dikaryotic genome assembly, especially for those complex genomes. Nucleus-by-nucleus strategy can be one of good choices for this kind of assembly.*

**Q2:** "Accurate phasing of these two nuclear genomes and their interactions have long been of interest but remain mostly unknown"

This is arguably false. Significant work that produced fully phased genome data and analyses from dikaryotic genomes that are even larger than the one analyses here have been published, including in this journal.
See H Duan et al. 2022, EC Henningse et al 2022, J Sperschneider et al 2021 B Schwessinger et al. 2021 to name a few...

***Response:*** *The sentence "Accurate phasing of these two nuclear genomes and their interactions have long been of interest but remain mostly unknown" was revised into "Accurate phasing of these two nuclear genomes and their interactions have long been of interest (Lines 22-23)".*

**Q3:** L92 (and elsewhere): In the present study, we used a "nucleus-by-nucleus strategy

"Please define. This is very vague terminology that is used multiple times in this paper but has never been defined. It sounds cool, but ultimately what the authors did is using monokaryon reference and HiFi and HiC data. How is this a nucleus-by-nucleus approach?

***Response:*** *Indeed, we assembled the dikaryon genome of Tr01 using monokaryon reference and HiFi and HiC data. Duo to the specific features of Tr01 genome, containing various heterozygosity regions and having several inter- and intra- chromosome rearrangements, we use contigs assembled by Hifiasm to generate one nuclear genome showing identical genomic architecture and high sequence similarity to monospore reference at first. We then use the remaining and common contigs to generate the other nuclear genome. So we called this approach as "nucleus-by-nucleus strategy".*

*“In the present study, we used a nucleus-by-nucleus strategy to assemble the dikaryotic genome of the sporeless T. fuciformis isolate Tr01. First, the complete haploid genome of one monospore isolate was generated as a reference genome, in which recombining regions were replaced by allelic sequences to assemble the complete genome of one nucleus. The remaining contigs and common contigs in low heterozygosity regions were then assembled from PacBio HiFi reads to assemble the other nuclear genome.” was revised into “We generated a high-quality* *genome sequences of a monospore isolate of T. fuciformis Tr01, and adopted a nucleus-by-nucleus strategy to sequence and assemble its dikaryotic genome: using the monospore genome as a reference, we assembled one nuclear genome showing identical genomic architecture and high sequence similarity to the reference at first, and generated the other genome using remaining and common contigs. (Lines 117-121)”*

**Q4:** L94: "in which recombining regions were replaced by allelic sequences to assemble the complete genome of one nucleus."

So you are creating new (potentially artefactual) assemblies ? This is very confusing for the reader. Please rephrase for clarity.

***Response:*** *We rephrased it. See also our response to question 3.*

**Q5:** L123: "We adopted a nucleus-by-nucleus strateg ".

This means nothing, really. Also, why using a convoluted and badly define approach instead of simply using HiFi to build scaffolds and HiC to obtain full chromosomes. HiFiasm has a MODE that uses Hi-C reads. This should be used here.

***Response:*** *See also our response to question 1.*

**Q6:** L145: "Hi-C analyses showed that contacts among chromosomes within the same nucleus were stronger than those between two nuclei":

need to show data/figure. In fact, authors should use the Nuclear Phaser pipeline from Daur et el 2022 Genome Biology to test this.

***Response:*** *We added a figure (Supplementary Figure 6) showing contact signals between Chr01 and other chromosomes.*

*“Hi-C analyses showed that contacts among chromosomes within the same nucleus were stronger than those between two nuclei.” was revised into “*NuclearPhaser[19] clustered all the *chromosomes into two sets, which were assigned to Tr01-Haplotype A and B nuclei, respectively. Chromosomes within the same nucleus had stronger Hi-C contact signals than those between two nuclei (Supplementary Figure 6).(Lines 186-189)”*

*“Hi-C signals are stronger among chromosomes within a nucleus than are those between nuclei. The assignment of chromosomes to each nucleus was achieved by assigning Chr01A to the Haplotype A genome then assigning other homologous chromosomes to either the A or B haplotype by comparing their Hi-C signals to those of Chr01A.” was revised into “Based on Hi-C chromatin* *contacts, chromosomal phasing in each nucleus was tested and corrected by* NuclearPhaser.(Lines 726-727)*”.*

**Q7:** L160-161:

This is sentence is confusing. How does telomere to telomere interactions suggests Hap1/2 are incorrect?

Please rephrase.

Also on this, any evidence for the presence of A/B compartments in this genome, as seen in Epichloe and more recently in AMF(Winter 2018., Yildirir 2022) ? Looking at your HiC maps, it looks like you have compartimentization ongoing.

***Response:*** *We rephrased “Although high levels of inter-chromosomal interaction arise due to telomere-telomere contacts in the T. fuciformis genome, analyses of Hi-C data verified the genome assemblies of Haplotypes A and B (Supplementary Figure 8)” into “For both Tr01-Haplotypes A and B genomes, each end of a chromosome showed strong contact signals with other chromosomal ends (Supplementary Figure 8). The telomere-telomere contacts provided another evidence for genome assembly verification. (Lines 207-210)”*

*Thanks for your suggestion on the presence of A/B compartments in this genome. We found: Hi-C contact signals among accessory compartments were stronger than those among core compartments. We marked regions showing Hi-C contact signals among accessory compartments in Supplementary Figure 3, and added the sentence “Hi-C contact signals among accessory compartments were stronger than those among core compartments (Supplementary Figure 3). (Lines 267-269)”.*

**Q8:** L218-220: The authors should determine the rate of rearrangements on these regions compared to the core genome, and see a significant increase is variability.

***Response:*** *All accessory compartments between two nuclear genomes are identical or near identical. The rate of rearrangements on the regions could not be determined.*

**Q9:** L294-296: These two paragraphs contain conclusive statements about how events may have occured. However, these are still speculations based on genomics and until empirical evidence is provided the authors should be careful in their interpretations and use "conditional" sentences explain these events.

I suggest the authors to use this part of the paper to build a model that explain what may have caused this event. A detailed figure explaining the model should be provided

L368: The results section contains a lot of discussion, so either mesh these into one results and discussions section or clearly separate both?

In any case, please make this much more concise.

***Response:***  *we revised the result section, and moved all the discussion parts of the result section into discussion section, including:*

1) *Replaced content in Lines 432-438 (old version) by content in Lines 254-276 (old version).*
2) *Contents of Lines 290-296 (old version), Lines 353-367(old version), Lines 443-459(old version), and Lines 466-482 (old version) were merged, and rephrased into content of Lines 559-601 (new version). In the rephrased paragraph, we proposed a model: unusual meiosis activities of the asymmetric dikaryotic genome lead to three peculiar features related to basidiospores of T. fuciformis Tr01, including few basidiospores generation, most monospore isolates bias to Haplotype A genome, and many monospore isolates carrying extra chromosomes. A detailed figure (Supplementary Figure 11) was provided to explain the model.*
3) *Combined the subsection “Extra chromosomes derived from meiosis of the T. fuciformis Tr01 dikaryon” into “New chromosomes in monospore isolates”, renamed as “Extra chromosomes in monospore isolates (Line 302)”. Combined the subsection “Genomic view of the basidiospore formation disorder in T. fuciformis Tr01” into “Genomic biases of T. fuciformis Tr01 monospore isolates”.*

*Model related to L294-296 (old version) was built. See Supplementary Figure 11C and Lines 592-601.*

**Q10:**L339-340: "With this strain, less production lossed due to basidiosporejection were reduced!"

Please rephrase.

***Response:*** *This sentence was moved to the discussion section. See Lines 621-622. “…massive production of spores can cause many serious problems, including respiratory allergic reactions to workers[**31**,* *32**] and mushroom production losses.”*

**Q11:** L376: "However, phasing of haplotypes within a dikaryotic genome still remains challenging."

yes, it is challenging but it is feasible with HiFi + HiC and the current paper doesn't demonstrate how their approach is better at achieving this.
***Response:*** *We removed the sentence “However, phasing of haplotypes within a dikaryotic genome still remains challenging.” , and revised a relevant part “In the present study, we used a nucleus-by-nucleus strategy to generate two haploid genomes for each nucleus of the dikaryotic* *basidiomycete T. fuciformis Tr01.” as “A Hi-C based phasing pipeline NuclearPhaser has been developed for dikaryon recently, and can be used to phase genomes of ~0.7 % heterozygosity with ~30-40× haploid genome coverage[**19**]. We used a nucleus-by-nucleus strategy to generate two haploid genomes for each nucleus of the dikaryotic basidiomycete T. fuciformis Tr01, which contains a large proportion of low heterozygosity regions (32.4 % of 10-Kb bins having < 0.7% heterozygosity). (Lines 458-464)”*

**Q12:** L389: "false positive fusions of allelic contigs that can result with current scaffolding methods[26]"

The authors actually never demonstrated that their phases are correct, and do not contain phase swaps for example. See Duan et al. 2022

***Response:*** *We revised “Our approach of accurately phasing the dikaryotic contigs based on the genome reference and recombination sites of offspring avoids false positive fusions of allelic contigs that can result with current scaffolding methods[**26**] .” as “Our approach accurately phased the dikaryotic contigs by determining if they were identical to the reference genome or not, avoiding false positive fusions allelic contigs of low heterozygosity that might result from current scaffolding methods[**19**,* *24**]. (Lines 475-479)”*

**Q13:** L432-438: no need to detail all the resuts again, Provide a good figure and legend that clearly explains this instead

***Response:*** *See response above. Content in Lines 432-438 (old version) was replaced by content in Lines 254-276 (old version). There is already one figure (Figure 7) for explanation.*

**Second round of review**

**Reviewer 2**

While I appreciate the authors' efforts to improve the quality and clarity of their manuscript, I feel the key issues raised by the reviewers have not been properly and sufficiently addressed, which considerably undermines the novelty of the study and weakens the robustness of the analyses.
Specifically, the authors claim that they assembled the genomes of the two nuclei in the dikaryotic Tremella fuciformis strain Tr01. However, I respectfully disagree. The haploid basidiospore, DBZ04, is a meiotic product, and thus, its genome cannot be the same as one of the two nuclei in Tr01 due to recombination and independent assortment of parental chromosomes during meiosis. Consequently, the two nuclear genomes they assembled cannot represent those of the two nuclei in Tr01.
The authors also analyzed additional basidiospores from Tr01, and correctly treated them as recombinants of the two parental nuclei. Thus, this begs the question as to why DBZ04 is analyzed differently. If DBZ04 is not unique in any way, then the study amounts to analyzing the genomes of a group of basidiospores, artificially choosing one, and assigning it as representative of the genome of one of the parental nuclei, which in my opinion is highly problematic. Such a study will also be considerably less novel as there have been many similar studies on a variety of fungal species.
Additionally, given the recombinant nature of DBZ04, it is actually surprising that the authors found evidence supporting its genome assembly from the Hi-C data of the dikaryon Tr01. Is the evidence statistically significant? Such analyses should be included.

**Reviewer 3**

I commend the authors for improving addressing many of my concerns, and for improving the clarity of their paper. However, there is still one main concern that the authors have overlooked.

In my original review, I mentioned that the authors did not provide evidence that their method is a improvement over the simple use of HiFi + HiC with HiFiasm (in HiC mode). The authors responded by stating that the heterozygosity of their model is too low to use the approach that many others successfully implemented to phase complete dikaryotic genomes, including massive and highly repeated ones.

Nevertheless, their comments are still not backed by data, simply by "unsustantiated claims"

As such, the authors have still do not responded to the relevant concern: if you make the claim that your new approach is better, then prove it or else use the approach everyone else is using.

I thus re-iterate that the authors should show the reader why using HiFi reads in combination with HiC and HiFiasm (In HIC mode) will lead to seemingl bad results. Furthermore, if it does results in bad assembly, how can the authors test if the quality of their HiC or HiFi data is not the cause of this?

Minor comments:

- The % of HiC reads showing miss-mapping as evidenced by Duan et al should be shown as a supplenntal Figure.
- Has the HiC model been implemented with HiFiasm?
- "Chromosomes within the same nucleus had stronger Hi-C contact signals than those between (Supplementary Figure 6)."
% of missmapped hic?
- ""contact signals among accessory compartments were stronger than those among core compartments (Supplementary Figure 3)".
Add supporting the presence of two dominant chromosome A/B compartments in filamentous fungi, as recently reported in "References".

**Authors Response**

**Point-by-point responses to the reviewers’ comments:**

**Reviewer#2**

**Q1:** Specifically, the authors claim that they assembled the genomes of the two nuclei in the dikaryotic *Tremella fuciformis* strain Tr01. However, I respectfully disagree. The haploid basidiospore, DBZ04, is a meiotic product, and thus, its genome cannot be the same as one of the two nuclei in Tr01 due to recombination and independent assortment of parental chromosomes during meiosis. Consequently, the two nuclear genomes they assembled cannot represent those of the two nuclei in Tr01.

***Response:*** *To address your concern of probable incorrect assembly of Tr01, we generated 40.3 Gb (713×) Oxford Nanopore ultra-long read sequences of Tr01 genome and selected 31,712 Nanopore ultra-long reads larger than 100 Kb, totaling 4.04 Gb (72×), to assist and verify Tr01 genome assembly. We conducted de novo genome sequencing using HiFi reads first and the bridged gaps using ultra-long reads without using DBZ04 genome as a reference.*

*PacBio HiFi reads of Tr01 were used for de novo assembly by HiFiasm in Hi-C mode, yielding two sets of phased contigs. One set was 30.3 Mb in size containing five complete chromosomes, and the other set was 30.2 Mb in size with seven complete chromosomes. Analyses of rDNA region in each genome set linked rDNA related contigs into one telomere to telomere (T to T) chromosome. Polished Nanopore ultra-long reads further were used to bridge gaps and assembled the rest 41 contigs of first genome set into six complete chromosomes, and the rest 46 contigs of second genome set into three complete chromosomes using telomeric repeat sequences as markers to reach T to T chromosome assemblies. After phased by* NuclearPhaser, 11 T to T chromosomes were clustered into *Tr01-Haplotype A genome, and the other 12* T to T *chromosomes in Tr01-Haplotype B genome. For chromosomes with heterozygosity between two haplotype genomes, each had significantly stronger Hi-C contact signals with chromosomes from the same nucleus than those from the other nucleus (P value = 3.9E-12, Supplementary Figure 4).*

***Assemblies of Tr01 were assessed by Nanopore ultra-long reads***

*Nanopore ultra-long reads were mapped to the Haplotype A and B genomes to verify the assembly. There were 7 TE insertions and 24 TE deletions in Haplotype A genome, and 5 TE insertions and 18 TE deletions in Haplotype B genome (Table 1-1, also Supplementary Table 2 in manuscript). These TEs were mainly composed of 16 copies of 683 bp LTR (30 %), 16 copies of 1239 bp LTR (30 %), and 7 copies of 6550 bp LTR (13 %). HiFi reads were mapped across each of the TE indels and verified that the sequences in the haplotypes A and B were correct, and all TE InDels. For example, two tandemly duplicated 6587 bp TEs were mapped* *and verified by 45 HiFi reads cross these two TEs, but the Nanopore Ultra-long reads mapped to this region contained three tandemly duplicated 6587 bp TEs. Samples for PacBio sequencing and Nanopore sequencing were collected in 2021 and 2023, respectively, 18 months apart. TE InDels occurred during this this period, separated by 12 rounds of vegetative reproduction, reflecting TE movement during mitoses in these 18 months. No chimeric contigs in Haplotypes A and B were detected, resulting from high accuracy and high redundancy of HiFi reads generated.*

Table 1-1. TE InDels detected in Nanopore ultra-long reads from mapping to Haplotypes A and B genomes

| Chromosome | TE insertions | TE deletions | Chromosome | TE insertions | TE deletions |
| --- | --- | --- | --- | --- | --- |
| Chr01A | 2 | 4 | Chr01B | 1 | 2 |
| Chr02A | 0 | 1 | Chr02B | 0 | 1 |
| Chr03A | 1 | 2 | Chr03B | 0 | 3 |
| Chr04A | 0 | 5 | Chr04B | 1 | 4 |
| Chr05A | 2 | 3 | Chr05B | 2 | 3 |
| Chr06A | 1 | 0 | Chr06B | 0 | 1 |
| Chr07A | 1 | 3 | Chr07B | 1 | 2 |
| Chr08A | 0 | 2 | Chr08B | 0 | 1 |
| Chr09A | 0 | 1 | Chr09B | 0 | 0 |
| Chr10A | 0 | 3 | Chr10B | 0 | 0 |
| Chr11A | 0 | 0 | Chr11B | 0 | 0 |
|  |  |  | Chr12B | 0 | 1 |
| Total | 7 | 24 | Total | 5 | 18 |

*Approximately half of the T. fuciformis Tr01 assemblies were assigned to either Tr01-Haplotype A or B genome, which are 28.15 Mb and 28.34 Mb in length, and contain 11 and 12 chromosomes, respectively.*

**Q2:** The authors also analyzed additional basidiospores from Tr01, and correctly treated them as recombinants of the two parental nuclei. Thus, this begs the question as to why DBZ04 is analyzed differently. If DBZ04 is not unique in any way, then the study amounts to analyzing the genomes of a group of basidiospores, artificially choosing one, and assigning it as representative of the genome of one of the parental nuclei, which in my opinion is highly problematic. Such a study will also be considerably less novel as there have been many similar studies on a variety of fungal species.

***Response: DBZ04 haploid genome were used for detecting recombination events during meiosis.***

*We agree that DBZ04 is a meiotic product of Tr01. The usefulness of this haploid genome is to detect recombination events during meiosis. All 11 chromosomes of DBZ04 shared high sequence collinearity with Tr01 Haplotypes A. Sequence similarity was 99.99% between these two genomes, except four regions in DBZ04 genome: Chr01:5177740-6011183, Chr05:1881594-2385495(3’end), Chr06 and Chr07:1662638-2272366(3’end), whereas these four regions shared more than 99.99 % identity with corresponding regions in Tr01-Haplotypes B genome. As a result, four homologous recombination breakpoints were detected in DBZ04 genome, including a double crossover in chromosome 1 and two single crossover in chromosomes 5 and 7 (Figure 1-1). Chromosome 6 is identical with Haplotype B. Chromosomes 4, 9, 10, and 11 are indistinguishable between haplotypes A and B genomes. The recombination breakpoints were also verified by mapping of Nanopore ultra-long reads as correct (Supplementary Table 3).*

*Figure 1-1. Sequence source distribution of DBZ04. Information of Chr04, Chr09, Chr10 and Chr11 was not included duo to near identical sequences between two nuclear genome of Tr01 leading to undetectable recombination events in the regions.*

***Novel findings from analyses of Tr01 basidiospores***

*Here are three novel findings from analyses of Tr01 basidiosores:*

1. *Although many studies on a variety of fungal species have been done to explore the recombinants of the two parental nuclei. However, few papers report the recombination behaviors between two parental nuclei with asymmetric genomes (Haplotype A has 11 chromosomes, and Haplotype B has 12 ones).*
2. *Genomic sequences of most basidiosores have strong bias to one of its parental genome (Haplotype A).*
3. *Recombination of two parental nuclei of Tr01 tends to result in formation of new chromosomes.*

**Q3:** Additionally, given the recombinant nature of DBZ04, it is actually surprising that the authors found evidence supporting its genome assembly from the Hi-C data of the dikaryon Tr01. Is the evidence statistically significant? Such analyses should be included.

***Response:*** *We counted the Hi-C contacts ratio of Haplotype A-B of each chromosome. The values of Haplotype A chromosomes ranged from 2.69 to 3.73, whereas values of Haplotype B chromosomes were from 0.24 to 0.34. The difference was highly significant, with P value of 3.9E-12 (ANOVA). The analyses were included in Supplementary Figure 4 and Lines 152-154 of manuscript.*

**Reviewer#3**

**Q1:** I commend the authors for improving addressing many of my concerns, and for improving the clarity of their paper. However, there is still one main concern that the authors have overlooked.

In my original review, I mentioned that the authors did not provide evidence that their method is a improvement over the simple use of HiFi + HiC with HiFiasm (in HiC mode). The authors responded by stating that the heterozygosity of their model is too low to use the approach that many others successfully implemented to phase complete dikaryotic genomes, including massive and highly repeated ones.

Nevertheless, their comments are still not backed by data, simply by "unsustantiated claims"

As such, the authors have still do not responded to the relevant concern: if you make the claim that your new approach is better, then prove it or else use the approach everyone else is using.

I thus re-iterate that the authors should show the reader why using HiFi reads in combination with HiC and HiFiasm (In HIC mode) will lead to seemingl bad results. Furthermore, if it does results in bad assembly, how can the authors test if the quality of their HiC or HiFi data is not the cause of this?

***Response:*** *To address your concern, we generated 40.3 Gb (713×) Oxford Nanopore ultra-long read sequences of Tr01 genome and selected 31,712 Nanopore ultra-long reads larger than 100 Kb, totaling 4.04 Gb (72×), to assist and verify Tr01 genome assembly. We conducted de novo genome sequencing using HiFi reads first and the bridged gaps using ultra-long reads without using DBZ04 genome as a reference. We no longer use the approach of reference-based genome assembly.*

*PacBio HiFi reads of Tr01 were used for de novo assembly by HiFiasm in Hi-C mode, yielding two sets of phased contigs. One set was 30.3 Mb in size containing five complete chromosomes, and the other set was 30.2 Mb in size with seven complete chromosomes. Analyses of rDNA region in each genome set linked rDNA related contigs into one telomere to telomere (T to T) chromosome. Polished Nanopore ultra-long reads further were used to bridge gaps and assembled the rest 41 contigs of first genome set into six complete chromosomes, and the rest 46 contigs of second genome set into three complete chromosomes using telomeric repeat sequences as markers to reach T to T chromosome assemblies. After phased by* NuclearPhaser, 11 T to T chromosomes were clustered into *Tr01-Haplotype A genome, and the other 12* T to T *chromosomes in Tr01-Haplotype B genome. For chromosomes with heterozygosity between two haplotype genomes, each had significantly stronger Hi-C contact signals with chromosomes from the same nucleus than those from the other nucleus (P value = 3.9E-12, Supplementary Figure 4).*

***Assemblies of Tr01 were assessed by Nanopore ultra-long reads***

*Nanopore ultra-long reads were mapped to the Haplotype A and B genomes to verify the assembly. There were 7 TE insertions and 24 TE deletions in Haplotype A genome, and 5 TE insertions and 18 TE deletions in Haplotype B genome (Supplementary Table 2). These TEs were mainly composed of 16 copies of 683 bp LTR (30 %), 16 copies of 1239 bp LTR (30 %), and 7 copies of 6550 bp LTR (13 %). HiFi reads were mapped across each of the TE indels and verified that the sequences in the haplotypes A and B were correct, and all TE InDels. For example, two tandemly duplicated 6587 bp TEs were mapped and verified by 45 HiFi reads cross these two TEs, but the Nanopore Ultra-long reads mapped to this region contained three tandemly duplicated 6587 bp TEs. Samples for PacBio sequencing and Nanopore sequencing were collected in 2021 and 2023, respectively, 18 months apart. TE InDels occurred during this this period, separated by 12 rounds of vegetative reproduction, reflecting TE movement during mitoses in these 18 months. No chimeric contigs in Haplotypes A and B were detected, resulting from high accuracy and high redundancy of HiFi reads generated.*

**Q2:** The % of HiC reads showing miss-mapping as evidenced by Duan et al should be shown as a supplenntal Figure.

"Chromosomes within the same nucleus had stronger Hi-C contact signals than those between (Supplementary Figure 6)."
% of missmapped hic?

***Response:*** *We revised the figure of Supplementary Figure 6 (Now Supplementary Figure 4). In the revised figure, we showed the percentage of Hi-C contacts to Haplotype A and B for each chromosome. For chromosomes in Haplotype A genome, Hi-C reads contacts to Haplotype B represent the miss-mapping part. All the data were generated by NuclearPhaser.*

*In Supplementary Figure 4 legend, “Inter-chromosomal interactions between Chr01A /Chr01B and each of other chromosomes. Y-axis, contact signal values.” was revised into “Genome assembly of Tr01 was supported by Hi-C analyses. Y-axis, percentage of Hi-C* *contacts to Haplotype A (dark orange) and B (dark blue) of each assembled chromosome. Numbers on top line represent Hi-C contacts ratio of Haplotype A-B of each chromosome. Each pair of Chr04, Chr09, Chr10 and Chr11 being identical except for some SVs, leading to their Hi-C contacts being irregular. Their data were not included in the Figure.”.*

Q3: Has the HiC model been implemented with HiFiasm?

***Response:*** *In the assembly of current genomic version, we used HiFiasm in Hi-C mode to phase two haplotype genomes by Hi-C contact signals.*

Q4: ""contact signals among accessory compartments were stronger than those among core compartments (Supplementary Figure 3)".
Add supporting the presence of two dominant chromosome A/B compartments in filamentous fungi, as recently reported in "References".

***Response:*** *There are already 5 references (Ref 19, 21, 23, 24 and 25) listed in paragraph 2 of section Discussion to support the presence of two dominant chromosome A/B compartments in filamentous fungi. We added another two recent ones: Ref 20 and Ref 22.*

**Third round of review**

**Reviewer 3**

I commend the authors on a strong revision. Happy with the latest revisions.

**Reviewer 4**

I was tasked with reviewing this revised version of the manuscript to assess whether the authors have adequately addressed the concerns raised by Reviewer 2.

Overall Assessment:
I find that the authors have successfully addressed the concerns of Reviewer 2. The revisions made to the manuscript are substantial and resolve the issues previously pointed out.

Specific Comments:

I concur with Reviewer 2's initial concerns regarding the representation of the genomes of the two nuclei in the dikaryotic Tremella fuciformis strain Tr01. The authors have now rectified this issue by generating a new analysis that includes the Tr01 genome. They used a combination of HiFi, HiC, and nanopore technologies to build a contiguous dikaryotic genome assembly. Furthermore, they employed HiC/Nuclear phaser to separate the assembly into the two nuclear genomes. This approach effectively addresses the concerns about using a meiotic product (DBZ04) as a representative genome. Correctly now the DBZ04 haploid genome is utilized to study recombination events during meiosis.

Minor Edits:

Line 289: The text states "nine chromosomes," but it should read "eight" as Hap A has 11 chromosomes.
Line 425: Change "Duo" to "Due."

In conclusion, the authors have made significant improvements to the manuscript, and I believe it is now ready for publication, pending the minor edits mentioned above.

**Authors Response**

**Point-by-point responses to the reviewers’ comments:**

**Reviewer#4**

**Q1:** Line 289: The text states "nine chromosomes," but it should read "eight" as Hap A has 11 chromosomes.

***Response:*** *Changed as suggested.*

**Q2：**Line 425: Change "Duo" to "Due."

***Response:*** *Changed.*
